# Membrane rigidity regulates *E. coli* proliferation rates

**DOI:** 10.1038/s41598-022-04970-0

**Published:** 2022-01-18

**Authors:** Samuel Salinas-Almaguer, Michael Mell, Victor G. Almendro-Vedia, Macarena Calero, Kevin Carlo Martín Robledo-Sánchez, Carlos Ruiz-Suarez, Tomás Alarcón, Rafael A. Barrio, Aurora Hernández-Machado, Francisco Monroy

**Affiliations:** 1 Centro de Investigación y de Estudios Avanzados, Unidad Monterrey, Vía del Conocimiento 201, PIIT, 66600 Apodaca, NL Mexico; 2 Departamento de Química Física, Universidad Complutense de Madrid, Av. Complutense S/N, 28040 Madrid, Spain; 3 Translational Biophysics, Instituto de Investigación Sanitaria Hospital Doce de Octubre (IMAS12), Av. Andalucía S/N, 28041 Madrid, Spain; 4 ICREA, Pg. Lluís Companys 23, 08010 Barcelona, Spain; 5 Centre de Recerca Matemàtica, Edifici C, Campus de Bellaterra, 08193 Bellaterra, Barcelona Spain; 6 Departament de Matemàtiques, Universitat Autònoma de Barcelona, 08193 Bellaterra, Barcelona Spain; 7 Barcelona Graduate School of Mathematics (BGSMath), Barcelona, Spain; 8 Instituto de Fisica, U.N.A.M., Apartado Postal 20-364, 01000 Mexico, D.F. Mexico; 9 Departament Fisica de la Materia Condensada, Facultat de Fisica, Universitat de Barcelona, Diagonal 645, 08028 Barcelona, Spain; 10 Institute of Nanoscience and Nanotechnology (IN2UB), Universitat de Barcelona, Barcelona, Spain

**Keywords:** Biophysics, Membrane biophysics

## Abstract

Combining single cell experiments, population dynamics and theoretical methods of membrane mechanics, we put forward that the rate of cell proliferation in *E. coli* colonies can be regulated by modifiers of the mechanical properties of the bacterial membrane. Bacterial proliferation was modelled as mediated by cell division through a membrane constriction divisome based on FtsZ, a mechanically competent protein at elastic interaction against membrane rigidity. Using membrane fluctuation spectroscopy in the single cells, we revealed either membrane stiffening when considering hydrophobic long chain fatty substances, or membrane softening if short-chained hydrophilic molecules are used. Membrane stiffeners caused hindered growth under normal division in the microbial cultures, as expected for membrane rigidification. Membrane softeners, however, altered regular cell division causing persistent microbes that abnormally grow as long filamentous cells proliferating apparently faster. We invoke the concept of effective growth rate under the assumption of a heterogeneous population structure composed by distinguishable individuals with different FtsZ-content leading the possible forms of cell proliferation, from regular division in two normal daughters to continuous growing filamentation and budding. The results settle altogether into a master plot that captures a universal scaling between membrane rigidity and the divisional instability mediated by FtsZ at the onset of membrane constriction.

## Introduction

The emergence of new strains of bacteria resistant to current antibiotic treatments constitutes a health-care problem worldwide^1–3^. Resistant variants appear as an unavoidable consequence of natural selection under the evolutionary pressure imposed by the generalized use of antibiotics^4–6^. Antibiotic strategies are mostly based on attacking specific biochemical targets^7^, or in creating membrane pores that shortcut the membrane potential^8^. The adaptative resistance of bacteria includes biochemical mechanisms for antibiotic inactivation, target modification, reprogramed permeability, and bypass of metabolic pathways^9,10^. However, despite the accumulated knowledge on bacterial biochemistry and the large amounts of resources invested to develop new drugs, bacterial resistance is still on the rise^11^. Determination of new biophysical mechanisms and coupled pathways for antibiotic resistance will complement current therapeutic strategies regarding combined usage of antibiotics with different mechanisms in different situations of bacterial infection. Therefore, unconventional antibacterial strategies must be explored, particularly if mutagenic bacterial populations can be confronted with new scenarios to which they are not adapted^11,12^.

In this work, we focus on the mechanical characteristics of the bacterial membrane as a possible target to regulate colony proliferation. In Gram-negative organisms such as *E. coli*, the outer lipid membrane is entailed by a thin peptidoglycan (PG) layer^13^, which is dynamically linked to the inner lipid bilayer^14,15^. The divisional machinery exploits the prokaryote protein FtsZ^16^, which dynamically polymerizes together with more than a dozen of accessory proteins as contractile filaments in a ring-shaped divisome placed on the cytosol side of the lipid bilayer^17–19^. The Z ring operates critically at highly regulated FtsZ level^20,21^, under tight control of the cell shape during the cell cycle and proliferation^22,23^. In a biochemical crosstalk with the outer PG synthesis apparatus^24,25^, and a mechanical concert with the inner lipid bilayer^26,27^, the PG-synthase/FtsZ-treadmilling divisome is known to exert the membrane remodelling forces involved in prokaryote proliferation^24–28^. Furthermore, all bacteria support a lipid homeostasis that regulates the mechanical properties of their lipid membranes enabling them to thrive in a wide range of environments^29,30^. Indeed, metabolic recycling of lipid intermediates is known to be crucial for membrane stability in bacteria^28–32^. Specifically, *E. coli* is known to modulate its lipid composition along the whole cell cycle, but especially during cell division^29^, a fact that could be argued as a biophysical cytokinetic control through the extrinsic regulation of the mechanical properties of their lipid bilayers. Our hypothesis stands on the recent consensus that growth conditions can determine variable microorganism resources leading phenotype variations^28,33,34^. As evidence on such biophysical pathways of bacterial proliferation control over resistant phenotypes, the antipersister potency of membrane-active antimicrobial agents has been recently shown to correlate with their ability to increase membrane fluidity^35^.

**Figure 1:**
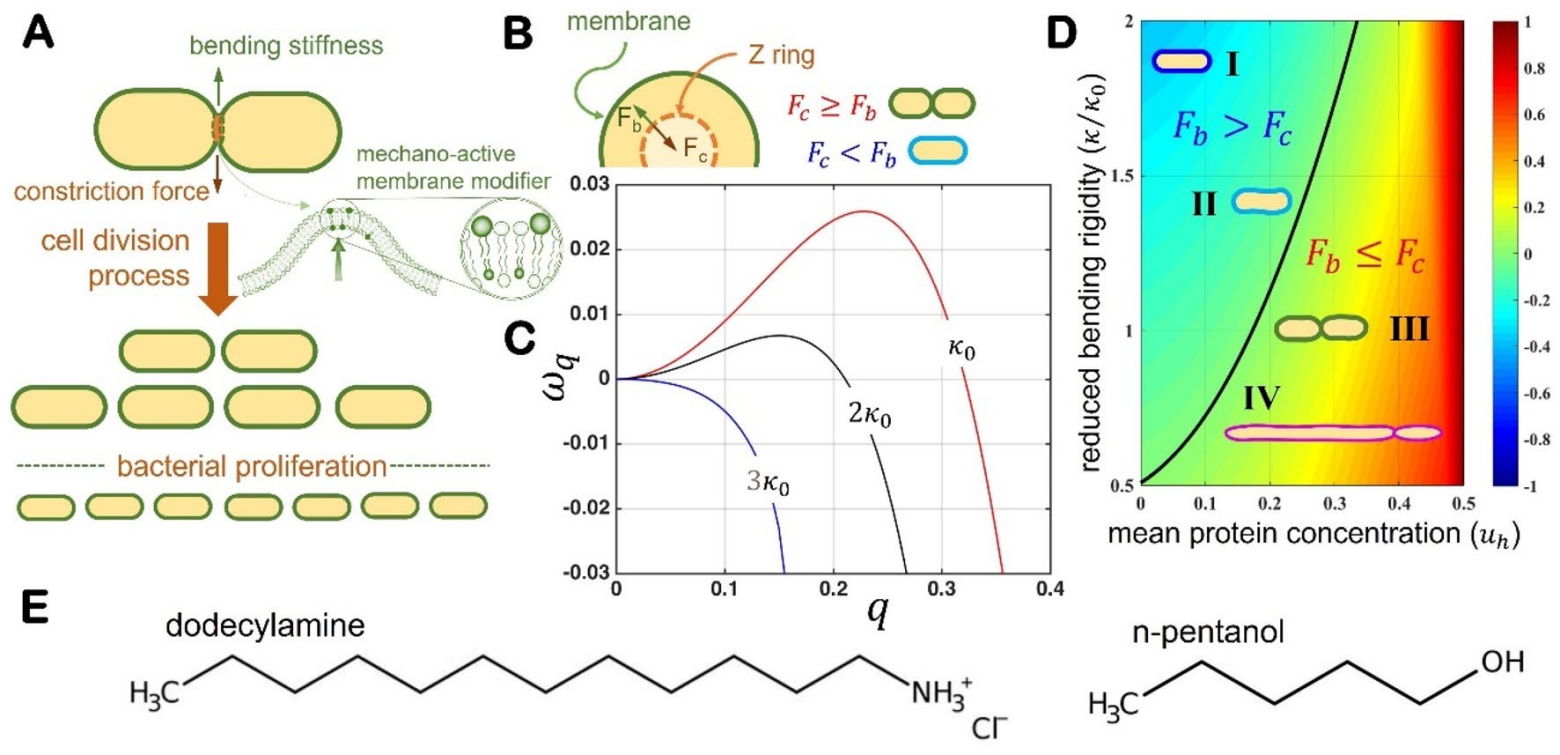
Biophysical rationale for mechanical control of bacterial proliferation. (**A**) Our model posits that by increasing the bending stiffness of the bacterial membrane, we can hinder the onset of the constriction associated with the formation of Z-rings competent in cell proliferation, as shown in panel (**B**) equatorial cell section with a detail of the force trade-off between the contractile force exerted by the Z ring and the opposing resistance exerted by the rigidity of the membrane. Whereas ${F}_{c}\ge {F}_{b}$ drives membrane constriction leading cell division, however at ${F}_{b}>{F}_{c}$ constriction is hindered thus cell proliferation stopped. (**C**) Linear mechanical stability captured by our theoretical model of bacterial membrane dynamics based on the membrane reorganization of FtsZ as competent rings^37^. Our analysis determines under which conditions small perturbations of an initial uniform FtsZ distribution decay. Mathematically, this is expressed in terms of the membrane mode exponential growth rate $Re\left[\omega \left(q\right)\right]$, which represents the rate at which a spatial perturbation with characteristic length ${q}^{-1}$ evolves in time. Depending on cell lengths, the divisional instability occurs at given low-$q$ modes in which membrane dynamics becomes unstable leading cell proliferation^37^. At given FtsZ concentration (in this plot, ${u}_{h}=0.3$), when $\omega \left(q\right)>0$ the associated perturbation grows exponentially as unstable modes (red line; corresponding to $\kappa \approx {\kappa }_{0}$). By contrast, increasing rigidity leads to decreasing instability corresponding to nearly stable membrane dynamics $\omega \left(q\right)\approx 0$, at hindered constriction (black line; $\kappa \approx 2{\kappa }_{0}$), and finally stable $\omega \left(q\right)<0$, flat interface in a nondividing cell (blue line; $\kappa \approx 3{\kappa }_{0}$). Our model predicts that changes in the bending stiffness can stop the onset instability from happening (at $\kappa \approx 3{\kappa }_{0}$). (**D**) State diagram with the different regions in parameter space; the black line separates membrane stable states (subcritical, ${F}_{b}>{F}_{c}$), and unstable regions leading to constrictional division (${F}_{b}\le {F}_{c}$): (I) Exhausted cells at high membrane rigidity (near-frozen) and subcritical FtsZ level ($\kappa \gg {\kappa }_{0}$ and ${u}_{h}\ll {u}_{crit}$); (II) Rigid cells at hindered constriction ($\kappa \ge {\kappa }_{0}$ and ${u}_{h}\approx {u}_{crit}$); (III) Normal cells at critical constriction ($\kappa \approx {\kappa }_{0}$ and ${u}_{h}\approx {u}_{crit}$); (IV) Abnormally dividing softened cells at overcritical FtsZ level ($\kappa \ll {\kappa }_{0}$ and ${u}_{h}\ge {u}_{crit}$). The temperature color scale indicates proliferation rates. For details regarding the mathematical analysis, see Supplementary Note N1. (**E**) Molecules used as membrane modifiers: (a) Saturated fatty dodecylamine (DDA) as a long chain membrane packer. (b) *n*-pentanol as a short chain disarranging agent.

Upon this biophysical perspective, we propose a mechanical strategy to modify bacterial proliferation upon extrinsic changes in membrane rigidity elicited by elastoactive additives that target the lipid bilayers as inclusion agents (see Fig. 1 for a depiction). We have considered two dissimilar membrane scenarios featured by a different degree of molecular disturbance, either by long-chain fatty molecules that cause bilayer ordering or by small hydrophilic molecules inducing disorder. We specifically considered dodecylamine (DDA) as a compacting agent causing membrane stiffening and pentanol as a disordering additive that elicits membrane softening. By treatment with those and several other additives, we have altered the membrane composition in living *E. coli* microbes, showing a readapted membrane mechanics in the single cells and, consequently an effectively variable growth rate in the bacterial colonies. Our analytic rationale has been designed as follows. To infer individual proliferation and possible phenotypic variation under antibiotic mechanical stress, we investigated bacterial counts and morphological cytometry in the colonies. The kinetic growth parameters of colony proliferation were also determined by conventional turbidity measurements. By exploiting a cutting-edge method for the analysis of the membrane fluctuations^36^, we then measured the effective bacterial stiffness as determined in vivo at the single-cell level. The experimental results reveal a critical scaling relationship between proliferation rates and bending membrane rigidity. In a physical approach to the force-generating automatisms underneath we built upon the mechanical theories of bacterial cell division^37–40^. We have implemented a nonlinear setting for the FstZ-driven constriction field in the single cells^40^, which predicts a variety of FtsZ containing individuals proliferating with a heterogenous population dynamics. Our biophysical approach (see Fig. 1A,B), which exploits theoretical methods combined with experiments performed in vitro and in vivo (see Supplementary Notes N1–N5), has got insight on the extrinsic regulation of the mechanical properties of the bacterial lipid bilayers as a tuneable pathway to interfere with the normal process of membrane constriction that masters cell division. The method potentially hinders bacterial proliferation in an unconventional way that could help to design novel antibiotic strategies upon the physical automatisms of cell membrane mechanics.

## Results

### Membrane rigidity model of hindered cell constriction

In order to analyse the nonlinear mechanism underlying cell divisional control under membrane rigidity, we exploit a mathematical model of its mechanical role on determining the membrane instability that leads membrane constriction^37^. The relevant details regarding our model are given in the Supplementary Materials (see Supplementary Note N1). It belongs to the Canham-Helfrich (CH) class governed by effectively linear curvature-elasticity [see Eqs. (S1)–(S4)], and accounts for the contractile forces acting nonlinearly on the cellular membrane^41^. Membrane dynamics is described by kinetic equations of the Cahn–Hilliard type [see Eqs. (S5)–(S6)]. Linear stability analysis predicts that enough increase in membrane rigidity could allow us to control the onset of cell division (see Fig. 1C). Specifically, for a given concentration of FtsZ protein per membrane site, ${u}_{h}$, which globally determines the spontaneous curvature of the cellular shape, we determined a critical value of the bending modulus ($\kappa \to {\kappa }_{crit}$), above which cell division is hindered by the rigidity of the membrane (see Fig. 1D). Inspired by this theoretical prediction, we tried to demonstrate experimentally the mechanical hypothesis that macroscopic growth rates in the colonies are nonlinearly controlled by membrane rigidity in the individuals.

Bacterial cell mechanics and the physical modelling of the mechanisms of membrane constriction has a long history^13,30,33–42^. A notable example is the early work by A. Koch, whose surface stress theory linked the cell shape during constriction to the cell wall growth and the surface tension of the cell envelope^14^. The bacterial cell wall is composed of inner cytoplasm lipid bilayer membrane, outer lipopolysacharide bilayer, and intermediate periplasm PG layer sandwiched in between^14^. The thin PG layer of Gram-negative bacteria is found ca. 8–10 nm depth, e.g., in *E. coli*^13^, whereas it is substantially thicker in Gram-positive (20 to 80 nm)^43^. This difference makes the *E. coli* cell envelope as a layered composite with a rigidity ruled by the more compact lipid bilayers^13,43^. Our CH-model for proliferating *E. coli* division considers the relevant mechanical ingredients for membrane constriction integrated all-in-one as an effective membrane rigidity ${K}_{eff}$, which regulates the bending stress involved in the constriction instability that leads cell division. The active constriction machinery is fuelled by the membrane driving FtsZ-protein field $u\left(K\right)$, which is completely regulated by the effective membrane rigidity in a closed loop that defines the physical automatism for critical constriction leading cell division at $K\le {K}_{crit}$ (see Supplementary Note N1). As a theoretical novelty, the regulatory network for the growth dynamics of heterogenous populations structured by FtsZ content has been further developed to describe the dynamical connections between this low-level membrane mechanics and the top-level proliferation kinetics (see Supplementary Note N5).

### The membrane-packer dodecylamine hinders *E. coli* proliferation

**Figure 2:**
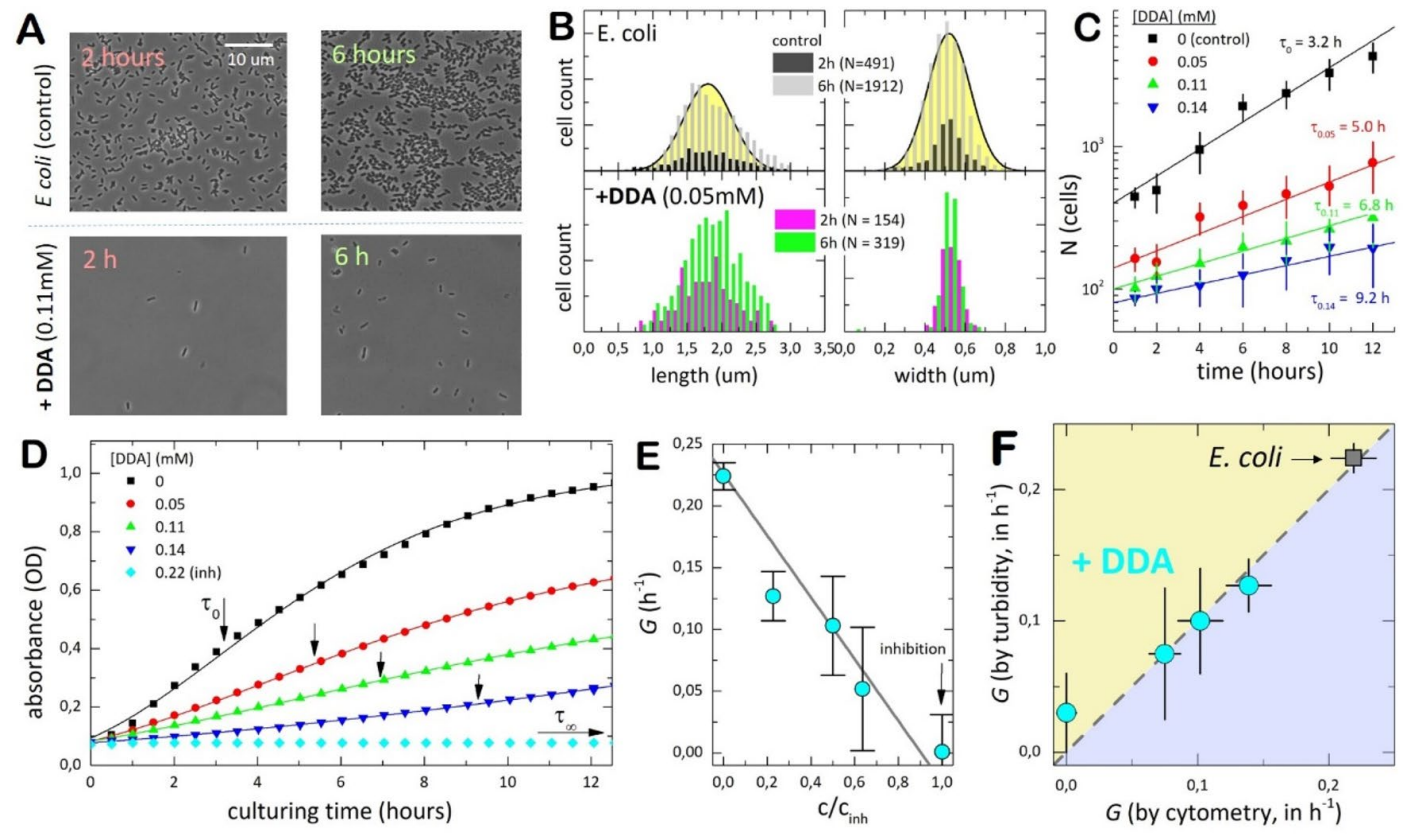
Growth inhibition by DDA. (**A**) Representative microscopy images of *E. coli* cultures suspended in nutrient medium at 37 °C for different conditions: (top panels) control, untreated *E. coli*; (down panels) added DDA at dose ${c}_{inh}/2=0.11\,{\mathrm{mM}}$ (half the inhibitory dose). Observations correspond to culture aliquots sampled in the exponential phase either at the beginning (after 2 h culturing time; left), or just before entering the stationary phase (after 6 h; right). A complete photoshoot at different conditions is shown in Suppl. Fig. S1. All experiments were performed in triplicate. (**B**) Cell counting with cytometric size specification for cultures as in (**A**) (SD normal distributions; $n>1000$ specimens). For untreated *E. coli*, we found (upper panels): *left*) cell length ${L}_{0}=1.8\,\upmu {\mathrm{m}}$ (${\sigma }_{L}=1\,\upmu {\mathrm{m}}$); *right*) cell width ${D}_{0}=0.52\,\upmu {\mathrm{m}}$ (${\sigma }_{D}=0.3\,\upmu {\mathrm{m}}$). Treatment with DDA does not change significantly cell dimensions; e.g. ${L}_{DDA}=1.9\pm 0.6\,\upmu {\mathrm{m}}$ and ${D}_{DDA}=0.54\pm 0.11\,\upmu {\mathrm{m}}$ at ${c}_{DDA}\approx 0.05\,{\mathrm{mM}}$ (lower panels). At least five different micrographs were analysed at each condition. (**C**) Kinetic cytometry. The cell counts $N\left(t\right)$ are monitored as culturing time progressed. Symbols represent averaged data from five different micrographs sampled at each time. The straight lines correspond to the best fits to the delayed exponential in Eq. (1); the values are the best fit doubling times that determine the specific growth rates ($G=ln2/\tau$). (**D**) Kinetic turbidity plots. The growth-curves are obtained as macroscopically averaged changes in optical density (OD) measured by plate turbidimetry. Data correspond to *E. coli* cultures treated with DDA-inhibitor at different reduced concentrations $c/{c}_{inh}$ increasing up to inhibition threshold [${c}_{inh}=0.22 \,{\mathrm{mM}}$ for DDA; symbols represent different concentrations specified in the legend; straight lines are the best fits to the logistic growth curve in Eq. (S12)]. Except for untreated *E. coli* (${t}_{0}=0.27\pm 0.04 h$), no evident lag times were detected in the presence of DDA (${t}_{0}\approx 0$). (**E**) Best fit values of specific growth rates ($G$) as a function of $c/{c}_{inh}$; approaching complete inhibition ($c\to {c}_{inh}$), we found $G\to 0$. (**F**) Correlation plot for growth rates measured from direct cell counting (*x*-axis) and turbidity measurements (*y*-axis).

Using quantitative cytometry, we featured cultures of *E. coli* treated in vivo with dodecylamine-hydrochloride (DDA), a long-chain/small-head hydrophobic agent potentially able to compact the lipid bilayer (as determined in vitro with model systems based on natural extracts of *E. coli* lipids; see Supplementary Note N2^44^). We considered *E. coli* MG1655 strain synchronously growing in nutrient LB broths at 37 °C (see “Methods” section and Supplementary Note N3). Due to the high bactericidal activity of DDA^45^, it was used at doses below a toxicity threshold for hampering cell mobility and complete growth inhibition (for DDA, ${c}_{inh}\approx 0.22 \,{\mathrm{mM}}$). Figure 2A shows micrograph shoots used for the cytometric study (more complete series are shown in Supplementary Fig. S1). Normal proliferation was observed in the control case of untreated *E. coli* microbes (Fig. 2A; top panel); they appeared with a normal spherocylindrical morphology characterized by averaged values of cell length ${L}_{0}=1.8\pm 0.5\,\upmu {\mathrm{m}}$, and cell width ${D}_{0}=0.52\pm 0.15 \,\upmu {\mathrm{m}}$ (see Fig. 2B; top panel), in agreement with literature data^46^. However, the cultures treated with DDA appeared clearly repressed (see Fig. 2A; bottom panel); the micrographs revealed a comparatively poorer cell count than in the control case with only a few bacteria in each observational field (see also Suppl. Fig. S1). No abnormal specimens were detected; all of them with a similar bacillus morphology and size as the untreated cells (see Fig. 2B; bottom). We analysed the synchronized cultures, as shown in Fig. 2C. In the exponential phase, after an induction period lasting a time ${t}_{0}$, the cell count $N\left(t\right)$ evolved in time as^47^:

1 $$N\left(t\right)={N}_{0}exp\left[G\left(t-{t}_{0}\right)\right],$$

in terms of the initial inoculum ${N}_{0}$, and the specific growth rate of the colony $G$, which corresponds in an homogenous population to an elemental doubling time for the dividing specimens ${\tau }_{p}=ln2/G$^47^. This equation describes a delayed exponential growth in the steady-state log phase $N\left(t\right)={N}_{0}^{\left(app\right)}exp\left(Gt\right)$, for which ${N}_{0}^{\left(app\right)}={N}_{0}exp\left(-G{t}_{0}\right)\le {N}_{0}$ represents an apparently reduced inoculum recovered after the lag phase^47,48^; only if ${t}_{0}=0$, then ${N}_{0}^{\left(app\right)}={N}_{0}$.

The untreated strain grew exponentially at a rate ${G}_{0}=0.22\pm 0.02 {h}^{-1}$ (${\tau }_{0}=3.2\pm 0.3 h$), without practically delay (${t}_{0}=0.3\pm 0.1 h\ll {\tau }_{0}$) (see Fig. 2C; upper curve), in agreement with literature^49^. Upon treatment with DDA, however, we observed a slowing-down associated to growth inhibition (see Fig. 2C). By reference to the control cultures, fewer cells proliferating at slower rates were observed with increasing the dose of DDA. For instance, at the highest DDA concentration studied, the doubling time was found longer than five times the reference value for untreated cells (${\tau }_{DDA}\approx 9 h$ at ${c}_{DDA}=0.14 \,{\mathrm{mM}}\ll {c}_{inh}\approx 0.22 \,{\mathrm{mM}}$). The lag times were found negligible (${t}_{0}\approx 0$ for *E. coli*/DDA). As a complementary piece on the hindering impact of DDA, Fig. 2D shows a kinetic analysis performed by turbidity measurements in culturing microplates (see “Methods” section). The kinetic plots featured similar inhibitory behaviour as the cytometric data with increasing DDA (see also Supplementary Fig. S2); particularly, short induction period, progressive slowing-down and decreasing saturation (see caption in Fig. 2D), in quantitative agreement with a previous report^45^. To get the kinetic parameters, we exploited an adapted form of Eq. (1) as the logistic curve in Eq. (S12), which accounts for saturation as a total biomass production at stationary plateau^48^. Figure 2E shows the fitted growth rates decreasing almost linearly with DDA concentration (also the cultured biomass (${Q}_{\infty }$); see Suppl. Fig. S2C), suggesting an inhibitory mechanism governed by a first-order kinetics; growth inhibition was indeed observed at ${c}_{DDA}\to {c}_{inh}\approx 0.22 \,{\mathrm{mM}}$ (see Fig. 2D; lower curve). As a quantitative proof of consistency, Fig. 2F points out the correlation between the growth rates measured by cytometry and turbidity (see also Suppl. Fig. S2). Altogether, the results evidenced progressive deceleration and further growth hindering induced by DDA in *E. coli*.

### The membrane-disordering pentanol alters bacterial proliferation as filamentous cells

**Figure 3:**
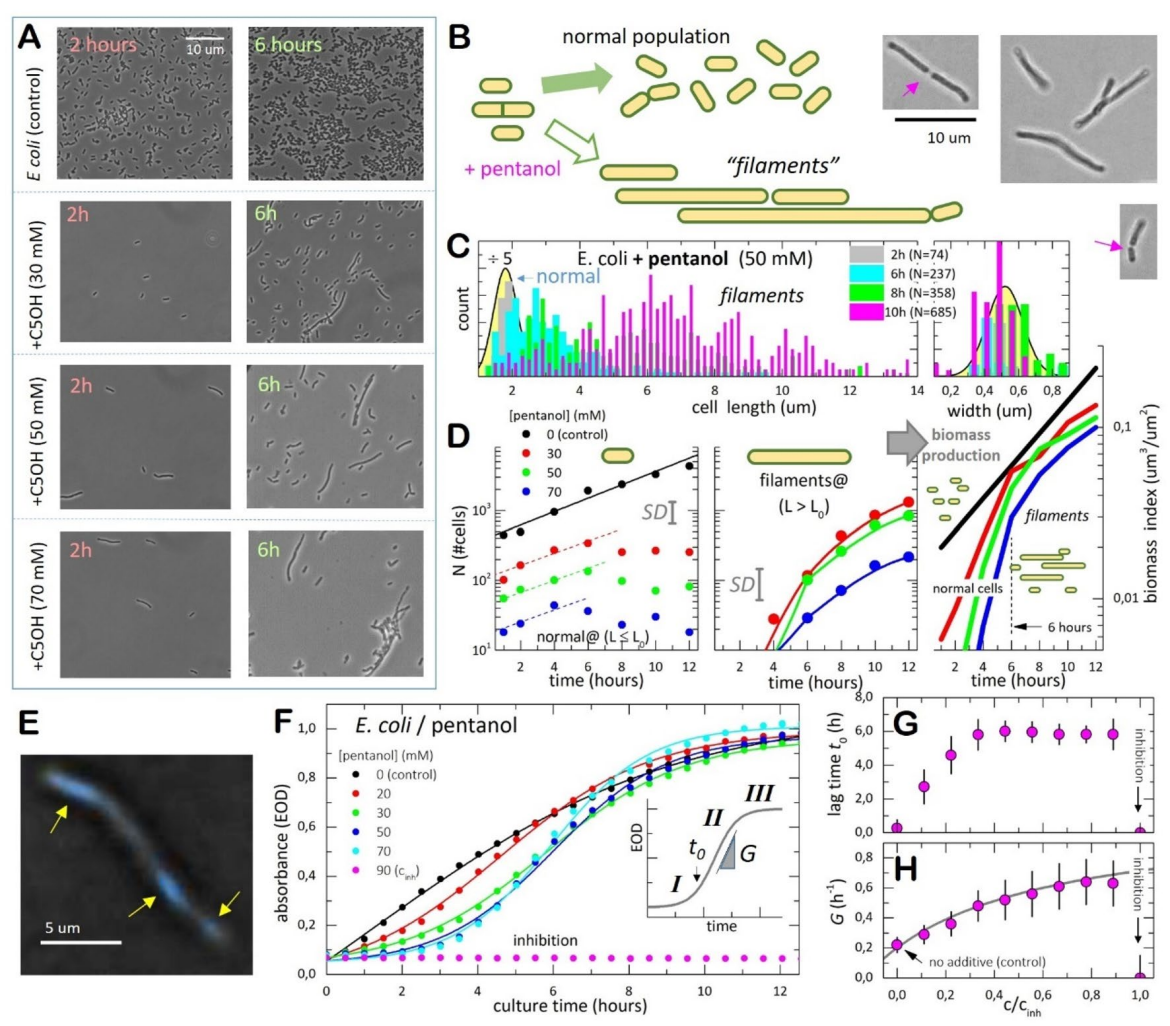
Abnormal proliferation under treatment with pentanol. (**A**) Representative micrographs of *E. coli* cultures supplemented with pentanol (C5OH) at the different concentrations specified (shoots at the same microscopy conditions as in Fig. 2): Control, untreated *E. coli* (top panels); added pentanol at variable doses (lower panels). (**B**) *Abnormal proliferation scenario:* in the presence of the membrane softener (pentanol), we observed anomalous bacterial growth as a marginal population of normal specimens coexisting with a prevailing population of bacterial persisters as long filaments that grow and eventually divide under elongation (see micrographs on the right; the arrows mark sites of division in the elongated filaments). (**C**) Cell counting with cytometric size specification for cultures treated with pentanol (as in **A**, five different micrographs were analysed at each condition; $n>100$ specimens): (left) cell length $L$; (right) cell width $D$. The normal size distributions for the untreated cells are included for comparison (yellowish Gaussians from Fig. 2B rescaled down dividing fivefold). (**D**) Cytometric kinetics by phenotype (at variable pentanol concentration as specified in the legend): (left panel) normal cells with length compatible with the canonical dimensions as determined for the untreated cells ($L={L}_{0}\pm {\sigma }_{L}$ with ${L}_{0}=1.9\,\upmu {\mathrm{m}}$ and ${\sigma }_{L}=1\,\upmu {\mathrm{m}}$); (central panel) filaments with an elongated cell length ($L>{L}_{0}+{\sigma }_{L}=2.9\,\upmu {\mathrm{m}}$). The right panel plots the time evolution of the produced biomass (at the different pentanol concentrations; same colour code). The biomass index is evaluated as the microbe coverage by all the bacterial specimens relative to the whole surface of the microscopy slides. (**E**) Zoomed fluorescence micrograph (×5) of a flaccid filamentous specimen representing typical bacterial persisters that proliferate longitudinally without division but with several nucleoids replicated along the filament (yellow arrows; DNA appears blue by Hoechst staining). (**F**) Kinetic turbidity plots (same experimental rationale and legend as in Fig. 2D but corresponding to heterogenous bacterial cultures including normal and filamentous cells). Three kinetic regimes of bacterial proliferation are clearly discernible: (I) induction phase (at $t<{t}_{0}$); (II) exponential phase; (III) stationary phase (at $t>{t}_{\infty }\approx 9h$) (see inset). The kinetic plots experience progressive delay in the induction phase and acceleration in the exponential phase upon increasing pentanol concentration (see legend). Complete growth inhibition is observed at ${c}_{inh}=90 \,{\mathrm{mM}}$ (magenta). The straight lines correspond to the best fits to the logistic curve in Eq. (S12) (in terms of the lag time ${t}_{0}$, and an effective growth rate $G$). The dependencies of the fitted parameters in terms of pentanol concentration appear in: (**G**) lag times; (**H**) effective growth rates (the straight line represents the best fit to the Monod’s law; see main text for details).

A different scenario occurred in cultures treated with pentanol (C5OH), a short-chain fatty alcohol chosen to be a fermentative metabolite with a high affinity for *E. coli* membranes at limited bacteriostatic toxicity (for C5OH, ${c}_{inh}\approx 8\,{\mathrm{g}}/{\mathrm{L}}\,\approx 90\,{\mathrm{mM}}$)^50^. This elastoactive molecule is enough hydrophobic to be soluble in the microbial lipid bilayers and quite hydrophilic at expanding interaction with the polar heads in these bilayers^50,51^. Indeed, the high affinity of pentanol for *E. coli* as a membrane softener relies on its ability to structurally distort the lipid packing, a fact already determined in vivo^52^, and here verified in vitro (see Suppl. Note N2). The micrographs in Fig. 3A evidenced a progressive poorer bacterial cell count after culturing *E. coli* with pentanol, followed by the appearance of abnormal specimens as elongated filaments (see also Suppl. Fig. S3), likewise in previous reports with similar additives acting as membrane disruptors^34,53^. This phenotypic adaptation resulted in a heterogenous population of normal cells coexisting with filamentous individuals; whilst only a few normal cells underwent regular division, some of such overgrown filaments were observed to further elongate whereas others eventually divide (see micrographs in Fig. 3B; right). Even at relatively low pentanol concentrations, the filaments constituted the prevalent subpopulation (e.g., at $c\approx {c}_{inh}/2\approx 50\,{\mathrm{mM}}$; see Fig. 3A, third panel). At higher inhibitory stresses, the normal cells are practically depleted (e.g., at $c=70\,{\mathrm{mM}}\,\approx 0.7{c}_{inh}$; Fig. 3A, lower panel). Figure 3C shows representative results from the cytometric analysis (for a more comprehensive dataset, see also Suppl. Fig. S3). After 2 h incubation, compared to the control case, we counted comparatively fewer cells with a relatively homogeneous size compatible with the normal status of the *E. coli* strain ($L{\approx L}_{0}\approx 2\,\upmu {\mathrm{m}}$ and $D{\approx D}_{0}\approx 0.5\,\upmu {\mathrm{m}}$; see the yellowish distributions in Fig. 3C). Lapsed 6 h under pentanol incubation, we found a similarly low count of normal cells but an increasing number of filamentous persisters. Longer culturing times lead to a prevalent abnormal cell proliferation into variably larger ($L>{L}_{0}$) and slightly slender ($D\lesssim {D}_{0}$) filamentous specimens^34,54^, which resulted in very broad distributions of cell lengths (see caption of Fig. 3C). The quantitative cell count is shown in Fig. 3D, which plots the number of specimens totalled at different pentanol concentration. We considered two phenotypes as stratified by cell length: normal cells (with $L\le 2.9\,\upmu {\mathrm{m}}=L_{0}+\sigma /2$; left panel), and large filamentous cells namely filaments (with $L\ge 3\,\upmu {\mathrm{m}}$; central panel). At short times, we recorded time-rising counts of normal cells decreasing with the pentanol dosed; here, the filaments count remains low. At long times, however, a stabilization of the normal population was detected (Fig. 3D; left), followed by an increase in the number of filaments (Fig. 3D; central panel). The biomass production was also estimated as a function of the culture time (Fig. 3D; right panel). Below the control case (monotonic increase), it resulted in a non-monotonic rise structured in two-stages: (1) an initial induction period at times below 6 h, corresponding to the initial expansion of the normal population (in equilibrium with death cells killed by the antibiotic), and (2) a consecutive saturating stage corresponding to further filament elongation. At high pentanol concentration ($c\ge 50\,{\mathrm{mM}}$), the filamentous persisters prevail as polynucleoid cells capable of tolerating the antibiotic action (see Fig. 3E)^50^.

### Apparent optical density in heterogenous populations upon pentanol treatment: effective proliferation rates

We opted by an alternative kinetic study of the global proliferation rates using turbidity measurements in culturing microplates (see “Methods” section). Because longer filaments scatter more light than normal cells^55^, we expected the changes in optical density accounting rather for cell elongation than for cell number growing^55^. In the following, we will refer to as an effective optical density (EOD), and the calculated proliferation rate as an effective growth rate (EGR), which integrates the proliferation velocity of elongating specimens as a global rate for biomass production^56^ (as accounted for by the Hills–Wright (HW) model of structured bacterial growth in heterogeneous populations^57^). Figure 3F shows the kinetic plots obtained at increasing pentanol concentration, which are evidenced as a logistic growth with three consecutive proliferation regions compatible with fittings to Eq. (S12) (see inset)^56,57^. The lag times were found to increase from ${t}_{0}\approx 0$, up to a ceiling value at relatively low pentanol dose (${t}_{0}^{\left(max\right)}\approx 6h$ at $c>0.3{c}_{inh}\approx 30\,{\mathrm{mM}}$; Fig. 3G); during the induction period, bacterial proliferation remains depressed by the pre-exponential factor in Eq. (1), this is ${N}_{0}^{\left(app\right)}/{N}_{0}=exp\left(-G{t}_{0}\right)$ with ${t}_{0}\gg 0$. In the exponential phase, the effective growth rates were observed increasing with pentanol concentration (see Fig. 3H). They display an inhibitory feature compatible with the Monod’s law; this is $G\left(c\right)/{G}_{max}\approx c/\left(K+c\right)$^47^. As compared to the reference value ${G}_{0}\approx 0.2 {h}^{-1}$ (for untreated *E. coli*), the maximal proliferation velocity is raised at relatively low concentrations of pentanol; ${G}_{max}\approx 0.7 {h}^{-1}\gg {G}_{0}$, as expected for a limiting metabolic substrate interacting at high affinity with the microbes ($K\approx 20\,{\mathrm{mM}}\ll {c}_{inh}$). The results demonstrate how bacteria metabolically adapt their growth characteristics to externally imposed conditions^33,34^.

### Effective membrane rigidity: membrane fluctuations in single cells by high-performance cell contour tracking

**Figure 4:**
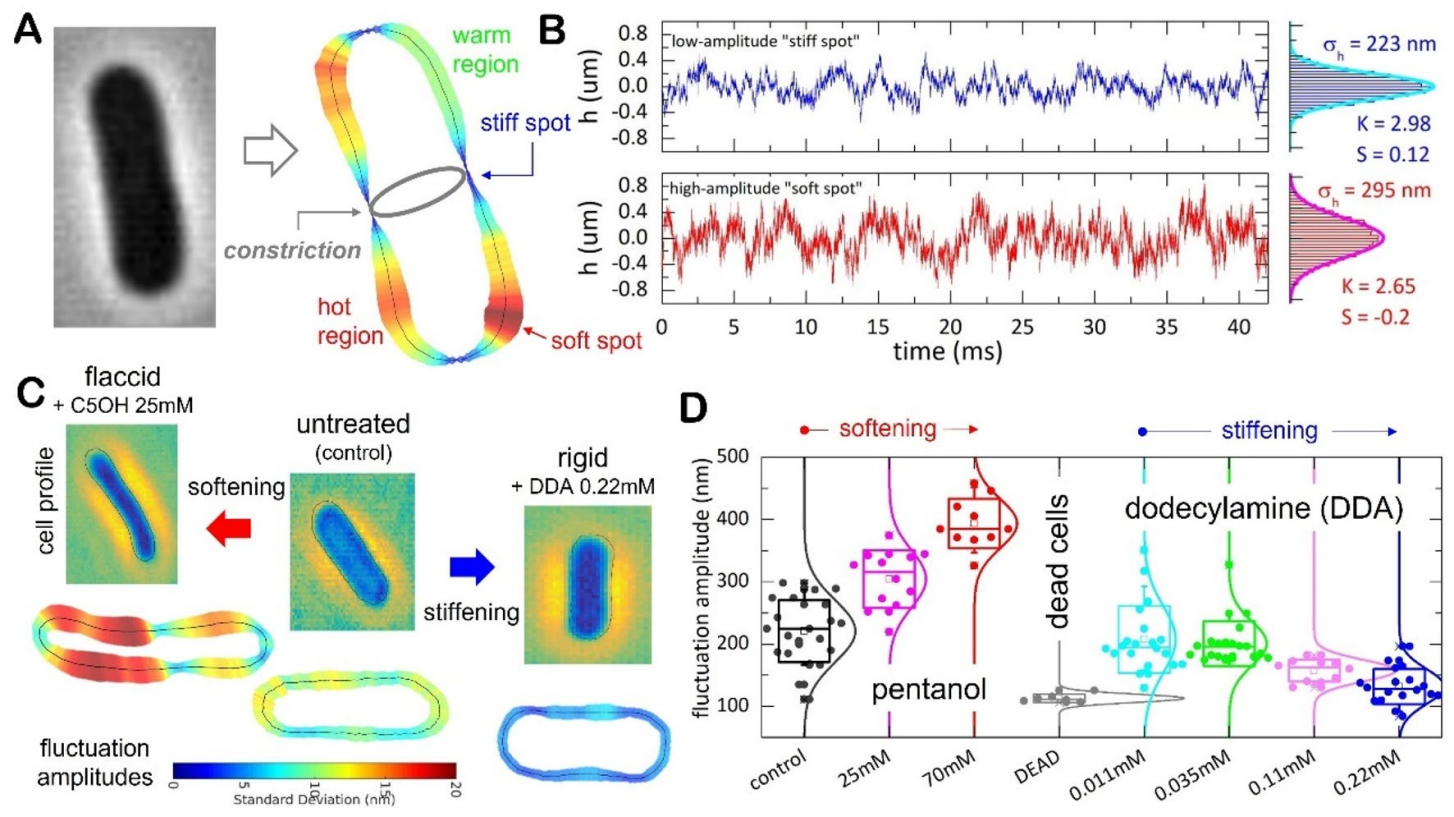
Single-cell membrane fluctuation spectroscopy as performed in *E. coli* (MG1655 strain) growing exponentially in a nutrient media at physiological temperature (37 °C). To record movies of the membrane fluctuations, single cells at pre-divisional status were observed under the optical microscope in the phase-contrast mode (see “Methods” section): (**A**) after tracking membrane fluctuations (see Suppl. Note N4), the bacterial profile is converted in spatial maps of dynamic properties estimated from the stochastic trajectories of every membrane emplacement, these are $h\left(x,t\right)$. The spatial map obtained represents the rms-value of the fluctuation amplitudes, $\Delta h\left(x\right)\equiv {\langle {h}^{2}\left(x,t\right)-{\langle h\left(x,t\right)\rangle }^{2}\rangle }_{t}$, which differentiates soft and rigid regions in a mechanically heterogeneous membrane (see main text for details). (**B**) Typical time series of membrane fluctuations $h\left(x,t\right)$; top panel) stiff membrane site undergoing low amplitude fluctuations, down panel) soft spot undergoing larger fluctuations. Depending on the mechanical status, fluctuation amplitudes of variable standard deviation (${\upsigma }_{h}$) distribute near-normally as characterized by skewness ($S$) and kurtosis ($K$) (right panels). Stiff spots normally behave as low-amplitude thermal noise (Gaussian-like; $S\approx 0$ and $K\approx 3$), and soft spots as high-amplitude, long-tailed trajectories with an out-of-equilibrium activity ($S\approx 0$ and $K>3$). (**C**) Fluctuation analysis in the modified cells upon treatment with membrane elastoactive agents (false colour images): left panels) membrane stiffening by DDA; central panels) untreated *E. coli* cell; right panels) membrane softening by pentanol (see main text for details). (**D**) Single-cell statistics for the variance of the fluctuation amplitudes ($\Sigma$). Data sampling box plots in relevant cases ($N>10$); statistical significance is defined as differences on the fluctuation amplitudes with respect to the null hypothesis (control: untreated cells; significance $99\%$). The box size indicates SD, and the whiskers the significance band including outliers. In order to better visualization, the normal distributions are included as straight lines. Different biological situations are indicated in the legends.

As a further step on demonstrating a cell mechanical control, we tested on the membrane fluctuations by exploiting a previous method of cell contour segmentation at sub-pixel resolution^36^, which was adapted to analyse cylindrical cells using high velocity videomicroscopy (see Supplementary Note N4). This high-performance tracking methodology overcomes possible artifacts arising from limited resolution^36,58^; see “Methods” section. As depicted in Fig. 4, we exploited this membrane fluctuation spectroscopy to study single specimens of *E. coli*. Figure 4A shows the spatial distribution of the amplitude fluctuations $h\left(x\right)$, as mapped for a wild-type specimen captured when constriction started up (see Supplementary Movies); all the experiments were performed at this reference state as represents a metabolically active pre-divisional status^47,59^. We detected spatially heterogenous maps revealing hot regions of intense membrane fluctuations in coexistence with warmer zones (see Fig. 4A; right panel). For the sake of example, Fig. 4B shows two time series recorded, respectively, at a membrane emplacement of small amplitudes assumedly a “stiff spot” (top panel), and at a more “active” site with larger and volatile fluctuations, assumedly a “soft spot” (down panel; see caption for details). The division site was observed always “stiff” (see Fig. 4A), as corresponds to a highly tensioned membrane emplacement under the contractile action of the FtsZ divisome^37^.

As a phenotypic descriptor, we considered the spatial average of the fluctuation variance calculated over the whole cell contour; this is $\Sigma \equiv {\langle {\sigma }_{h}\left(x\right)\rangle }_{x}$. For untreated *E. coli*, we measured ${\Sigma }_{0}=220\pm 20\,{\mathrm{nm}}$ (see Fig. 4D; central panel). The CH-theory relates membrane fluctuations inversely to effective membrane stiffness as ${\Sigma }^{2}/A\simeq {k}_{B}T/K$ (relative to the membrane area $A$)^41^; by assuming a cylindrical bacterial area $A\approx \pi DL\approx 9 \,{\upmu {\mathrm{m}}}^{2}$ (for a cell diameter $D\approx 1 \,\upmu {\mathrm{m}}$, and length $L\approx 3 \,\upmu {\mathrm{m}}$), we estimated ${K}_{0}\simeq {k}_{B}T\left(A/{\Sigma }_{0}^{2}\right)\approx 200 {k}_{B}T$, on the same order of magnitude than the flexural rigidity measured by optical tweezers^60^, or under hydrodynamic deformability in microfluidic platforms^61^. Aligned with previous works in softer biomembranes^36,62,63^, these fluctuation features supported our cell contour tracking method at adequate performance. Once the single-cell fluctuation platform was validated for *E. coli*, we analysed the mechanical impact of the membrane modifiers (see Fig. 4C).

### The membrane fluctuations are modified by elastoactive additives: effective membrane stiffness

Inspired on our pivotal theoretical prediction about a rigidity-controlled cell division and steered by the above evidence on a proliferation altered by elastoactive agents, we took steps forward to demonstrate their impact as membrane modifiers in vivo (as far an effective change of membrane stiffness $K$ is elicited). Figure 4C shows the experimental results obtained in a single-cell basis. We systematically mapped heterogeneous distributions of membrane fluctuations, which emerged as a functional organization of the “living” membranes to exert spatiotemporally regulated remodelling forces^13,64^. Arguably, these (out-of-equilibrium) membrane motions are driven by the cytokinetic apparatus^13^. Therefore, we expect the amplitudes varying as ${\Sigma }^{2} \sim F/K$, where $F$ represents the strength of the local cytokinetic stress against the effective rigidity of the membrane. In our antibiotic test bench, pentanol and DDA were detected to modify the reference rigidity ${K}_{0}=200\pm 20{k}_{B}T$ (see Fig. 4C; central panel), with an evident impact on the observable fluctuations (lateral panels). On the one hand, the presence of pentanol caused generalized membrane softening as revealed by a boosted appearance (“softer membrane”, since ${\Sigma }_{C5OH}>{\Sigma }_{0}$, then ${K}_{C5OH}<{K}_{0}$; see Fig. 4C, left panel). These extensive hot regions underwent non-Gaussian fluctuations as corresponded to the enhanced activity by cytokinetic machineries (see Supplementary Fig. S4); the progressive softening appeared compatible with the flaccid aspect of the elongated cells (see Fig. 3). On the other hand, the cells treated with DDA experienced a pronounced reduction of the membrane fluctuations, which became homogeneously feeble with respect to the untreated cells (see Fig. 4C; right panel). Contrarily to the softening action of pentanol, the DDA-modified cells appeared, in general, with colder (thermalized) membrane fluctuations (“stiffer membrane”, since ${\Sigma }_{DDA}<{\Sigma }_{0}$, then ${K}_{DDA}>{K}_{0}$). Such a progressive stiffening gave rise to apparent cellular exhaustion (see Supplementary Fig. S5). Despite the natural variability between specimens, these qualitative differences were statistically significant as supported by quantitative variations with respect to the control cases (see Fig. 4D). In the rigid limit of dead cells ($K\approx {K}_{crit}\gg {k}_{B}T$), one expects ${\Sigma }_{min}^{2}\approx {F}_{min}/{K}_{crit}\to 0$, where ${F}_{min}\approx {k}_{B}T$ constitutes the lowest fluctuation ground (the null hypothesis for testing the active fluctuations in the living cells)^37^. As a relevant evidence, the fluctuation observables have enabled a quantification of the mechanical impacts caused by dissimilar elastoactive agents at work in the bacterial membranes (see Supplementary Movies). The effective changes in membrane rigidity could determine the differences in proliferation dynamics observed in the living cells under the antibiotic stresses caused by these substances.

### The effective membrane stiffness is controlled by the modified lipid bilayers

**Figure 5:**
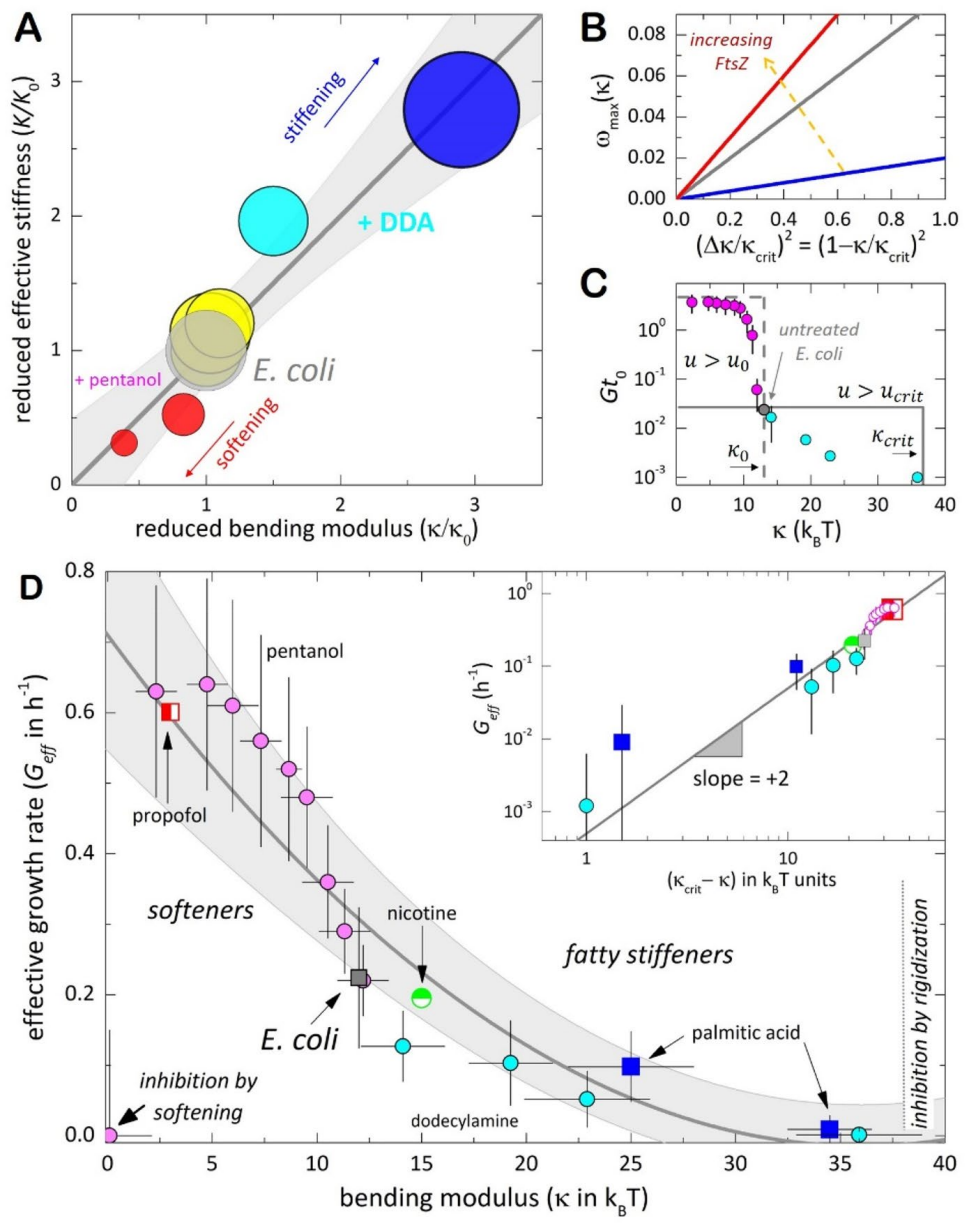
Universal impact of mechanical membrane modification on *E. coli* proliferation dynamics. (**A**) Correlation between the actual membrane rigidity in the living cells (Fig. 4E) and the bending modulus of the lipid bilayers in the vesicle models (Suppl. Note. N2). The reduced values of the effective membrane stiffness $$K/{K}_{0}$$ (*y axis*), and of the bending modulus $$\kappa /{\kappa }_{0}$$ (*x axis*), are calculated by reference to the unaltered *E. coli* system ($${K}_{0}=102 {k}_{B}T$$ for the real cells; $${\kappa }_{0}=12 {k}_{B}T$$ for GUVs made of *E. coli* lipids). The circular areas represent SDs from the single-cell data (accounting for cell variability; variances in the GUVs’ data are considerably smaller). The straight line plotted in grey represents the correlation diagonal. The shadowed region is the confidence band for the linear fit at full correlation ($$p<0.05)$$. (**B**) Rate of the critical mode (first unstable mode at $${\omega }_{max}$$), as numerically calculated for different FtsZ concentrations close to the critical line for membrane instability in the CH-model of membrane dynamics developed in Suppl. Note N1 (black line in Fig. 1D); legend: $${u}_{h}=0.23>{u}_{crit}$$ (blue line); $$0.26$$ (grey); $$0.30$$ (red). The quadratic dependence $${\omega }_{max}\sim {\left({\kappa }_{crit}-\kappa \right)}^{2}$$ at variable FtsZ-concentration reveals the key role of the rigidity order parameter $$\Delta \kappa \equiv {\kappa }_{crit}-\kappa$$ in describing the onset of criticality. Consequently, the exponential rate $${\omega }_{max}$$ is expected to directly fix the effective proliferation rate in an universal way determined by $${G}_{eff}\sim {\omega }_{max}\sim {\Delta \kappa }^{2}$$. (**C**) Proliferation lag argument $$G{t}_{0}$$ as obtained from turbidity kinetics in terms of membrane stiffness (values of $$\kappa$$ for the corresponding lipid bilayers). The normal physiological status is found at unaltered bending rigidity ($${\kappa }_{0}$$) and regulated FtsZ level ($${u}_{0}$$); symbols: untreated *E. coli* (grey), pentanol-treated (magenta), DDA-treated (cyan). *Existence boundaries:* Criticality at $${u}_{h}>{u}_{crit}$$ and $$\kappa <{\kappa }_{crit}$$ (solid); FtsZ overload at $${u}_{h}>{u}_{0}$$ and softened membrane at $$\kappa <{\kappa }_{0}$$ (dashed). The onset of criticality starts as a normal proliferation for untreated *E. coli* at vanishing lag time i.e., at $${t}_{0}\approx 0$$ near $${\kappa }_{0}$$. Complete growth inhibition is asymptotically reached at $$\kappa \mapsto {\kappa }_{crit}>{\kappa }_{0}$$ and $${u}_{h}\mapsto {u}_{crit}$$ (see text for details). (**D**) Effective growth rates of the proliferating cultures of *E. coli* under treatment with different membrane elastoactive agents (*softeners:* pentanol, propofol; *stiffeners:* DDA, nicotine and palmitic acid). Symbols are experimental data obtained from turbidity measurements ($${G}_{eff}$$), corresponding to systems with an equivalent membrane rigidity ($$\kappa$$), as measured in the modified *E. coli* lipid bilayers (values from Suppl. Note N2). The experimental rates decrease quadratically as the bacterial membrane becomes stiffer. The results for all the different systems accumulate around a master plot $${G}_{eff}\sim {\left({\kappa }_{crit}-\kappa \right)}^{2}$$ (grey line: best fit; shadowed region: confidence band at $$99\%$$ confidence level). Growth inhibition occurs either at $$\kappa \to 0$$ (inhibition by softening instability), or at $$\kappa \to {\kappa }_{crit}$$ (inhibition by stiffening exhaustion). Near-universal proliferation dynamics is proven in agreement with theoretical prediction despite some data deviate slightly up from this plot (probably due to their dynamic complexity not fully captured by our minimal model). The inset shows the algebraic scaling in the double-log data plot $${G}_{eff}\sim {\Delta \kappa }^{\beta }$$, in strong support of quadratic scaling along the experimentally accessible range spanning almost more than three decades in time upon varying membrane rigidity by almost a hundred ($$\beta =2.1\pm 0.2$$).

A closed demonstration of this hypothesis calls to further experiments checking for the correlation between the membrane stiffness detected in the living cells ($K$), and the bending rigidity of the constituting lipid bilayers ($\kappa$). As a model in vitro, we used giant unilamellar vesicles (GUVs) made of *E. coli* lipid extracts. We took advantage of fluctuation spectroscopy to determine $K$ in the real cells and $\kappa$ in the GUV models (see “Methods” section). To get insight on the packing state of the modified lipid membranes, we additionally considered compression rheology in Langmuir monolayers. This complementary study of membrane rheology is included as Supplementary Materials (Suppl. Note N2). As a reference value for model *E. coli* lipid bilayers with a thickness $d\approx 5\,{\mathrm{nm}}$^63^, we measured a bending modulus ${\kappa }_{0}=12\pm 2 {k}_{B}T$^44,63^. For Gram-negative bacteria, in general, the effective membrane stiffness should scale as ${K}_{eff}/\kappa \approx {\left(D/d\right)}^{2}$^65^; being $D$ the cell wall thickness (including the hydrated periplasmic PG gel), and $d$ the single lipid bilayer depth. Since the native *E. coli* membrane is fourfold thicker than one single lipid bilayer i.e., $D=21\pm 3 \,{\mathrm{nm}}\,\approx 4d$^66^, we estimate ${K}_{0}\approx 16\kappa \approx 200 {k}_{B}T$, in quantitative agreement with the effective stiffness measured in vivo for the real bacterial membrane. Figure 5A shows the linear correlation between $\kappa$ and $K$. As a proof of concept, “mechanical treatment” with pentanol and DDA caused the same apparent impact in the living cells as their direct inclusion into the corresponding lipid bilayers. This validating result demonstrates the effectiveness of elastoactive bilayer insertion as a successful strategy for in vivo membrane rigidity regulation.

### Heterogenous population dynamics

As a necessary requirement for the integrative connection between membrane stiffness and growth rates, we developed a theory of bacterial proliferation for heterogenous populations (see Suppl. Note N5), which accounts for the FtsZ-mediated cytokinetic forces as implicit in our mechanical membrane model (see Suppl. Note N1). Once assumed heterogenous individuals at variable FtsZ concentration, effective cell proliferation arose from the constriction instability as predicted for the unstable membrane mode (see Fig. 1B). Above a critical FtsZ level ($u>{u}_{crit}$), we supposed normal proliferation arising from the divisional trade-off from competent Z-rings exerting enough contractile force against membrane rigidity (${F}_{c}\ge {F}_{b}$; as depicted in Fig. 1B). Below the upper rigidity threshold (${\kappa <\kappa }_{crit}$), this occurs at the maximal rate ${\omega }_{max}\left[u\left(\kappa \right)\right]$ (see Fig. 1D). In terms of the control parameter for the membrane instability ($\Delta \kappa {=\kappa }_{crit}-\kappa$), our heterogenous dynamics predicts exponential growth at an effective rate varying quadratically as ${G}_{eff} \sim {\omega }_{max} \sim {\Delta \kappa }^{2}$ (see Fig. 5B). Implicitly, this $u\left(\kappa \right)$-stratified proliferation is damped by the fraction of successful cell division [as fixed by the parameter $\alpha \left(\kappa \right)\le 1$; see Eq. (S18)]. For the reference elasticity state (${\kappa \approx \kappa }_{0}$), at regulated FtsZ levels ($u\approx {u}_{0}$), our theory describes normal colony proliferation at exponential growing (without delay), as given nearby the physiological doubling time [from Eq. (S20) for $\alpha =1$ and $u={u}_{0}$, one deduces ${G}_{0} \sim {\tau }_{p}^{-1}$ and ${t}_{0}\approx 0$]. Our theory also captures cases of unsuccessful cell division leading to proliferation delays [$\alpha <1$; see Eq. (S14)], which induce finite lag times, i.e. ${t}_{0} \sim {\left[log\left(2\alpha \right)\right]}^{-1}>0$ [see Eq. (S20)]. As a relevant connection with experiment, Fig. 5C shows the critical abolition of the lag phase in approaching the onset of normal proliferation (at $\kappa \approx {\kappa }_{0}$). Because abnormal FtsZ levels could induce significant delay (as observed in the colonies treated with pentanol; see Fig. 3G), our model considers repressed proliferation during a lag phase; thus ${t}_{0}\gg 0$ in softened cells under cell elongation ($\alpha \ll 1$ for $u>{u}_{0}\gg {u}_{crit}$; see legends in Fig. 5C). Uplifting FtsZ expression is indeed known to favour delayed cell elongation^21^, being a crucial resistance adaptation factor, for instance, in filamentous *E. coli* persisters subjected to shifts in temperature^67^, or with an altered lipid composition leading to bilayer disorder^68^. Stiffer membranes could also require higher FtsZ concentrations to constrict ($u>{u}_{crit}$ at $\kappa >{\kappa }_{0}$), leading structurally normal ($\alpha \approx 1$), although dynamically slower cell proliferation (${G}_{eff}{t}_{0} \to 0$ at $\kappa \to {\kappa }_{crit}$), as observed in *E. coli* after treatment with DDA (see Fig. 5C). The observed cues are pretty much compatible with a heterogenous population dynamics structured by FtsZ levels, which depicts an antibiotic scenario conceived as a mechanical constraint to the cell division mechanism; at $u>{u}_{crit}$, normal proliferation occurs only if ${F}_{c}\ge {F}_{b}$ at $\kappa \approx {\kappa }_{0}<{\kappa }_{crit}$ (see the existence boundaries in Fig. 5C). However, at $\kappa >{\kappa }_{crit}$ and/or $u<{u}_{crit}$ then ${F}_{b}>{F}_{c}$, thus normal cell division becomes no longer possible.

### Universal connection between membrane dynamics and proliferation rates: the effective growth rates are determined by membrane rigidity

The predicted instability onset was shown in Fig. 5C varying quadratically with the defective bending rigidity $\Delta \kappa ={\kappa }_{crit}-\kappa$ (as parametrized with respect to the upper limit for growth inhibition ${\kappa }_{crit}$). Such mechanical universality is pointed out by Fig. 5D, which collects all the experimental results with pentanol and DDA (and complementary data for propofol, nicotine and palmitic acid; see Suppl. Fig. S6 and Suppl. Table T1); the quadratic law of the proliferation rates was found as ${G}_{eff}\left(\kappa \right) \sim {\left({\kappa }_{crit}-\kappa \right)}^{2}$, with the critical membrane rigidity at ${\kappa }_{crit}=36\pm 5 {k}_{B}T$ ($\approx 3{\kappa }_{0}$, as estimated for the modified *E. coli* lipids; corresponding to ${K}_{crit}\approx 3{K}_{0}$ in the real cells). Likely, the predicted quadratic scaling was evident over almost four decades (see inset).

The universally quadratic law $G \sim {\Delta \kappa }^{2}$ appears as a physical connection between the proliferation kinetics (mediated by membrane FtsZ at supercritical levels $u>{u}_{crit}$), and the bending rigidity of the lipid bilayers (at $\kappa <{\kappa }_{crit}$). All the conditions studied for *E. coli* lye along a master plot that represents the critical domain of Fig. 1D (${F}_{c}\ge {F}_{b}$), in which cell proliferation occurs at rates determined by the membrane instability that fosters the cells to divide. Whereas the untreated cells constitute a physiological status belonging to the critical line of existence (the normal state of cell proliferation), other states of cell proliferation are also possible alongside (in compliance with the same membrane instability that allows for constriction). The symmetry is broken only at the extremes of the critical line; either as an upper singularity caused by explosive softening (the constriction instability diverges at $\kappa \to 0$), or as an upper asymptotic limit caused by rigidity exhaustion (at $\kappa >{\kappa }_{crit}$). Beyond these limits, membrane constriction does not occur anymore; there, cell proliferation ends up defining the two mechanical regions for cell death under elastoactive treatment, either cell freezing under membrane stiffening at minimal $\Delta \kappa \to 0$ (i.e. at effective membrane stiffening $K\to {K}_{crit}\approx 3{K}_{0}$), or cell elongation under effective membrane softening at maximal $\Delta \kappa ={\kappa }_{crit}$ (i.e. at $K\to 0)$.

## Discussion

Additional to the cytosol lipid bilayer, Gram-negative bacteria (such as *E. coli*), possess an outer lipid bilayer, which sandwich a hydrated layer of cross-linked peptidoglycan (PG) filaments that form the periplasmic cell wall. However, Gram-positive bacteria devoid this outer bilayer although reinforced by a much thicker and compact PG layer (*B. subtilis* e.g.). In general, bacterial membranes are composite and stratified, exerting an effective resistance against cytokinetic forces^13^. Upon the subsidiary action of PG as a dynamically adaptable wall for the maintenance of cell shape^22,23^, lateral tensions and flexural rigidities are efficiently overcome by the FtsZ-based constriction engine that operates from the cytosol side of the inner lipid membrane^24,27^. These mechanical ingredients are minimally considered in the theory of bacterial division here implemented. To test mechanical control of membrane constriction upon lipid rigidization, we have conducted a series of experiments checking several membrane modifiers with an impact on the proliferation dynamics of *E. coli*. Extrinsic membrane regulation has been performed by inclusion agents with a high affinity for the lipid bilayer and a structural capacity to modify their rigidity, hence their molecular packing (as shown in the auxiliary study with model *E. coli* membranes). We have tested two classes of elastoactive modifiers, namely, membrane stiffeners e.g., fatty substances as dodecylamine and palmitic acid, and packing agents as nicotine well recognized as bactericidal agents. To distort the lipid bilayer with short chain hydrophilic molecules able to impact as membrane softeners^69^, we have also considered two bacteriostatic agents—pentanol and propofol, with a variable tolerance impact on the stressed phenotypes that appear viable to thrive in the presence of these lipid distorting molecules. For bacterial proliferation to happen under antibiotic stress conditions either in cell dividing phenotypes or in non-dividing resisters, adaptative variations occur naturally on the FtsZ-mechanistics that regulate the cell-cycle^10,70^, and the coordinated lipid metabolism that allows for cell growth^29,71^. Homeostatic FtsZ levelling and regulated membrane mechanics are both widely considered as the protagonist actors in the physiology of *E. coli* proliferation^14–30^. Despite the great advances in super-resolution microscopy^72^, the biochemical routes of active cytokinetic force production and reactive membrane rigidity remain however elusive. Nevertheless, FtsZ and membrane lipids continue to be the primary protein and the mechanical object most often targeted in the very prolific research field that keeps still at resolving this intriguing biological problem^73–75^. Alongside such vibrating research avenue, we have here contributed robust experimental evidence on the relevant membrane automatisms that regulate prokaryote cell proliferation in *E. coli*.

On the one hand, membrane stiffening as induced in *E. coli* cells by DDA, palmitic acid or nicotine has been revealed lowering the growth rate in the rigidified individuals thus decimating the colonies. This bactericidal class of growth inhibition is known for fatty substances not only in Gram-negative bacteria^50,76^, but also in Gram-positive^77,78^. Also well-known is the accumulation of inviable cells in cultures treated with bilayer compacters such as nicotine^79^, or caffeine^80^. Although this bactericidal action is classically explained as a lethal genetic effect of fatty compounds^76,81^, our experiments reveal causality between lipid compaction and decreased growth rates under effective membrane stiffening.

On the other hand, membrane softening by bilayer disrupters, such as pentanol and propofol, has been suggested as a direct cause for accelerating the proliferation dynamics in *E. coli*. Similar to bilayer damage by fluidization^82^, and/or lethal permeability upsurge^83^, our study with membrane softeners has shown that, after a long induction phase in which the microorganisms are striving against their bacteriostatic action, some resistant cells can elongate as filamentous persisters during the exponential phase of colony proliferation. Other short-chain alcohols e.g. benzyl alcohol^52^, ethanol, hexanol^84^, water soluble anaesthetics like propofol, lidocaine and others^69^, small hydrophilic molecules^85^, as well as molecular disrupters binding to phospholipid head groups^86,87^, etc. are known to distort the lipid packing providing enough membrane softening to lead inhibition of bacterial growth. Membrane disruption results in further delocalization of membrane proteins impairing multiple essential processes including respiration^88^, nucleotide synthesis^89^, FtsZ assembly^10,68^, and nucleoid segregation^68,90^, with the collateral effect to harm the cytokinetic apparatus so as to prevent and/or delay the membrane constriction need for normal cell division. This is probably why hydrophilic membrane softeners lead to abnormally filamentous phenotypes^91^, mostly as dormant bacterial specimens (non-dividing but still viable), in which antibiotic targets become adapted in tolerance^54,91,92^. Despite the limited capacity of these altered microbes to generate normal offspring (as here shown with *E. coli* after pentanol treatment), their abnormal proliferation has allowed us to demonstrate mechanical causality upon membrane softening; from bilayer disruption at the molecular level, through faster membrane instability in the single cells, up to initially delayed but apparently accelerated growth rates in the colonies due to the rapid elongation of the filamentous cells.

In a global sight, our combined results with hydrophilic membrane disruptors (softeners) and hydrophobic compactors (stiffeners) have pointed out the crucial role of membrane rigidity as the key regulator of bacterial proliferation at the onset of a physical criticality, which is inherent to the cytokinetic process of membrane constriction need for the cells to divide. Our analysis suggests that the mechanical response of the bacterial membrane, and its associated effects on cell division, exhibit a certain degree of universality rather related to membrane mechanics than to the biochemical details of the constriction apparatus. Aligned with the current wisdom on the mechanistic control of bacterial cell division^28–40^, the evidence raised at variable membrane rigidity points out a physical regulation of the bacterial proliferation rates at feedback automatism with the cell size as determined by the FtsZ content. As an outlook, the universal scaling here discovered in agreement with a comprehensive theory of nonlinear membrane constriction could lay the foundation of a rational theory of the biophysical regulation of bacterial proliferation via membrane modifiers. This novel property-function relationship constitutes a promising platform for testing new antibiotic candidates targeting bacterial membrane mechanics.

## Methods

### Chemicals

All chemicals were supplied by Sigma, including *E. coli* PLE from Avanti. Ultrapure water for preparing solutions and culture media was from a Milli-Q unit (UV, polypropylene filter 0.2 μm; conductivity lower than 18 MΩ cm. Merck-Millipore). Chemicals and solutions were sterilized in autoclave.

### Synchronized bacterial culture

*E. coli* MG1655 strain, stored at − 80 °C in 20% glycerol and Luria Bertani (LB) medium, was cultured on 1.5% agar-LB plates for 16 h at 37 °C and afterwards scraped to 2 mL LB falcon tubes. Aliquots of 200 μL was fourfold diluted using 10 mL of LB, remaining stirred in an incubator at 37 °C. Culture growing was monitored by measurements of optical density (OD) in a spectrophotometer (at 600 nm). The values between 0.2 and 0.3 OD were used to trigger the next dilution step. Once the fourth dilution has been completed, we assume all the bacteria remaining in the same stage of the exponential phase. For further details, see Supplementary Note N3.

### Membrane mechanical modification by membrane elastoactive agents

From the synchronized bacterial suspensions, 200 μL were mixed with 2.4 mL of LB medium containing different concentrations of the different elastoactive agents previously dissolved. These treated bacterial solutions (and a control one with just 2.4 mL of pure LB) were cultured at 37 °C under mild stirring. Aliquots of 200 μL were poured into a flat bottomed 96-well microplate. These multiplexed samples were not sealed in order to allow for adequate oxygenation of the bacterial cultures.

### Turbidity measurements

Growth curves were obtained using a spectrophotometer Multiskan GO Thermo Scientific in 96-well microplates under continuous shaking and at constant temperature of 37 °C. This device records the OD as a measurement of absorbance at 600 nm. Each culture was replicated five times in the same microplate, and every experimental condition was repeated by quintuplicate in independent experiments. The uncertainty in the averaged values of the experimental parameters was obtained as a standard deviation measured over the $N=25$ replica considered at each experimental condition. The experimental growth curves were quantitatively fitted to a logistic growth curve. As far turbidity provides a macroscopic average for the bacterial culture in suspension, this technique determines the cultured biomass in an indirect way (see Supplementary Note N3).

### Colony cytometry

Aliquots of 200 μL were taken from the synchronized incubating solutions. Cells were mildly adhered in the microscope coverslide covered with an agarose mattress. Phase contrast micrographs were captured with an oil immersion objective (40× Nikon Plan Appo) in an inverted microscope (Nikon TE2000). At least ten photographs were shoot at each condition. Cytometric analysis was performed using MicrobeTracker^93^, combined with MATLAB R2020a algorithm (The Mathworks Inc.) (see Supplementary Note N3).

### Single cell fluctuation spectroscopy: effective membrane rigidity

Stacks of consecutive single-cell images were recorded with an inverted microscope in the phase contrast mode (Nikon Eclipse Ti2, equipped with a 100 W TI-12 DH Pillar Illuminator, an LWD 0.52 collimator, 100× oil immersion objective -Plan ApoVC, NA 1.45, and Nikon motorized autofocus). We only considered specimens adhered to the agarose mattress thus preventing for spurious drift motions (cell translation and rotation). For maximal signal-to-noise ratio, movies of the membrane fluctuations were captured at high-acquisition velocity (2000 fps) during 2 s of tracking time with a FASTCAM SA3 camera (Photron), using a magnifying telescope that resulted in an effective pixel size of 50 × 50 nm^2^. These hardware conditions allow for optimal spatiotemporal accuracy at minimized acquisition readout assuring artifact unaffected fluctuation tracking^36,58^. The phase-contrast membrane contours are focused on the cell equatorial plane in which they were digitally segmented using a custom-made algorithm for high-performance determination of the maximal gradient of the optical halo (128 points; ca. 50 nm lateral resolution)^36^. Because *E. coli* cells appears quite dark with respect to the optically transparent medium, the phase-contrast halo is accurately defined as a diffraction (Airy) profile along the normal directions (Fig. 4A). The contour segmentation algorithm operates at interpolating the membrane diffraction profile at sub-pixel resolution (± 0.1px; ± 5 nm as the highest accuracy at the best segmentation performance), with refined correction of possible center-of-mass translation and cell rotation as inferred for the collective motion of, respectively, the contour barycenter and the whole membrane profile with respect to the average (fiduciary) cell shape^36^. For mechanical measurements in single cells, the ensemble-averaged PDFs of the membrane fluctuations were built from the normalized histograms of the membrane height displacements $h\left(x,t\right)$, estimated over a population ($n>10$, typically). For each point in the membrane profile, we calculated the variance ${\sigma }_{h}^{2}\left(x\right)\equiv {\langle {h}^{2}\rangle }_{t}-{\langle h\rangle }_{t}^{2}$ (as a time average ${\langle\, \rangle }_{t}$). The effective membrane rigidity was then inferred from the spatial average of the fluctuation variances, ${\Sigma }_{h}^{2}\equiv {\langle {\sigma }_{h}^{2}\rangle }_{x}$; specifically, as ${K}_{eff}\simeq A{k}_{B}T/{\Sigma }_{h}^{2}$ (see Supplementary Note N4). To determine the bending rigidity of the equivalent model membranes $\kappa \left(\to {K}_{eff}\right)$, we evaluated the shape fluctuations of GUVs as described by the CH-bending free energy considered in the limit of small curvature deformations^41^ (see Supplementary Note N2).

## Supplementary Information

Supplementary Information.

## Data Availability

Raw data are available upon request to the authors.

## Code availability

Informatic codes are available upon request to the authors.
