# Supplementary material for: Membrane rigidity regulates E. coli proliferation rates
Source: Sci Rep. 2022 Jan 18;12:933. doi: 10.1038/s41598-022-04970-0 (PMC8766614; doi:10.1038/s41598-022-04970-0)
Supplement: Supplementary file 1 — Supplementary Information. [file 41598_2022_4970_MOESM1_ESM.pdf]

SUPPLEMENTARY MATERIALS

Membrane rigidity regulates E. coli proliferation rates.

Samuel Salinas-Almaguer[1,2], Michael Mell[2], Victor G. Almendro-Vedia[2], Macarena Calero[2,3], Kevin Carlo Martín Robledo-Sánchez[1], Carlos Ruiz-Suarez[1], Tomás Alarcón[4,5,6,7], Rafael A. Barrio[8], Aurora Hernández-Machado[5,9,10,*] and Francisco Monroy[2,3,*].

1. Centro de Investigación y de Estudios Avanzados, Unidad Monterrey, Vía del Conocimiento 201, PIIT, Apodaca NL 66600, Mexico
2. Departamento de Química Física, Universidad Complutense de Madrid, Av. Complutense S/N, E28040 Madrid, Spain
3. Translational Biophysics, Instituto de Investigación Sanitaria Hospital Doce de Octubre (IMAS12), Av. Andalucía S/N, 28041 Madrid, Spain
4. ICREA, Pg. Lluís Companys 23, E-08010 Barcelona, Spain.
5. Centre de Recerca Matemàtica, Edifici C, Campus de Bellaterra, 08193 Bellaterra (Barcelona), Spain.
6. Departament de Matemàtiques, Universitat Autònoma de Barcelona, 08193 Bellaterra (Barcelona), Spain.
7. Barcelona Graduate School of Mathematics (BGSMath), Barcelona, Spain
8. Instituto de Fisica, U.N.A.M., Apartado Postal 20-364, 01000 Mexico D.F., Mexico
9. Departament Fisica de la Materia Condensada, Facultat de Fisica, Universitat de Barcelona, Diagonal 645, E-08028 Barcelona, Spain
10. Institute of Nanoscience and Nanotechnology (IN2UB), Universitat de Barcelona, Barcelona, Spain

*To whom correspondence should be addressed; E-mail: a.hernandezmachado@gmail.com , monroy@ucm.es

Table of Contents

Supplementary Movies. Complete collection of movies for E. coli single cell membrane fluctuation analysis.

Supplementary Note N1. Membrane rigidity model of hindered cell constriction: Phase field approach.

Supplementary Note N2. Rheological study with model E. coli lipid membranes: Bilayer GUVs and Langmuir monolayers.

Supplementary Note N3. Synchronized bacterial culturing.

Supplementary Note N4. Single cell tracking of membrane fluctuations in spherocylindrical bacteria: Mechanical maps.

Supplementary Note N5. Heterogenous population dynamics.

Supplementary Figure S1. E. coli cultures treated with dodecylamine-hydrochloride (DDA).

Supplementary Figure S2. Turbidity measurements of E. coli cultures treated with DDA.

Supplementary Figure S3. E. coli cultures treated with pentanol (C5OH).

Supplementary Figure S4. Kurtosis of the membrane fluctuation distributions in living E. coli: Softening treatment with pentanol.

Supplementary Figure S5. Kurtosis of the membrane fluctuations under cell exhaustion in E. coli.

Supplementary Figure S6. Growth curves for E. coli cultured with propofol, nicotine and palmitic acid.

Supplementary Table T1. Complementary data for propofol, nicotine and palmitic acid: Effective growth rate in cultured colonies and bending rigidity in GUVs.

Supplementary References

Supplementary Movies. Complete collection of movies for E. coli single-cell membrane fluctuation analysis.

To be found in the public repository:
https://doi.org/10.5281/zenodo.5236807

Supplementary Note N1. Canham-Helfrich membrane rigidity model of hindered cell constriction: Phase field approach.

We build upon a nonlinear phase-field model of the dynamics of shape remodelling in vesicle membranes with a superposed protein field susceptible to undergo phase separation under curvature changes [1]. Under initial conditions corresponding to a homogeneous protein distribution in a spherocylindrical

geometry (resembling an E. coli bacteria), we studied the time evolution of the vesicle shapes undergoing equatorial constriction. Such time-dependent spontaneous curvature is driven by the protein field, which represents the concentration of a cytokinetic substance that anchors with the membrane thus changing the local curvature and producing constriction. Under geometry-driven protein segregation in a nonlinear field of the Landau-Ginzburg class (entailed for adequate order parameters on the membrane protein concentration u), we predict a membrane constriction mediated by the action of a phase-segregated protein structure (protein-rich), which resembles the role of a Z ring in the bacteria. Phase-field approaches of this class have a long history of application to studying the dynamics of membrane deformations governed by bending rigidity [2,3,4], including surface finite elements numerical solutions in membrane systems with a high compositional complexity [5]. By focussing on the phase-separation dynamics as described by our phase-field model, here we study the kinetics of constriction at the onset of the criticality appeared as a trade-off between the driving action of the competent Z rings (leading constriction under sufficient protein concentration), and the opposing reaction from the rigidity of the membrane (see Figure 1 in the main text for rationale and results). We are describing below the relevant aspects that give rise to our minimal kinetic model of bacterial constriction resembling the scission process of E. coli cells undergoing division.

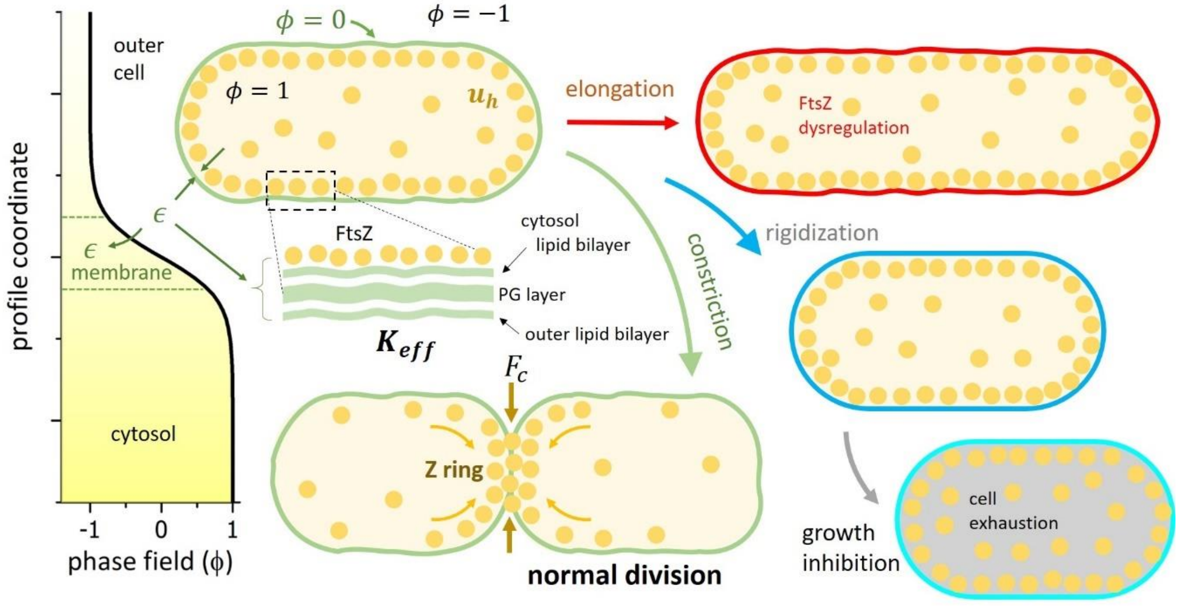

Figure SN1. Phase-field rationale for the membrane constriction by a cytokinetic apparatus based on the local accretion of spontaneous curvature under protein phase segregation (competent Z rings). Phase-field profile along a phase coordinate varying smoothly between the cytosol ($\phi = 1$) and the outer cell space ($\phi = -1$); in the hyperbolic tangent profile of thickness ϵ,

the equivalent membrane surface is placed at $\phi = 0$ (left panel). **System**: A bacteria-like vesicle is endowed with a thin flexible membrane ($\epsilon \to 0$), which coarse-grains all of its biological complexity in an effective stiffness K_{eff} that contains rigidities from the PG layer, lipid bilayers and membrane associated proteins (cytokinetic FtsZ and associated partners, among them). Cytokinetic proteins constitute a Landau-Ginzburg field susceptible to undergo phase segregation constituting Z rings at physiologically regulated protein level u_h. Both fields are mutually intercoupled in Eq. S1 through of the interactions between membrane elasticity and cytokinetic proteins as described in the field equations (Eqs. S2-S4). **Phase-transition scenarios:** ***Competent Z ring segregation leading membrane constriction and normal division)*** The cytokinetic protein symmetrically segregates in a ring-shaped domain with enough spontaneous curvature to constrict the membrane at mid-cell; this represents normal physiological conditions of membrane rigidity and regulated FtsZ levels in untreated E. coli cells. The constriction force F_c is given by the critical trade-off in the free energy functional at near-critical phase-segregating conditions (defining the criticality onset in Fig. 1D). ***Constrictional failure under membrane rigidization)*** The field of surface elasticity is too much rigid to enable curvature changes in the membrane thus no trade-off is stablished with the cytokinetic protein field, which remains homogenous at subcritical conditions; in vivo, excessive membrane rigidization leads to noncontractile, growth hindered conditions and eventual cell exhaustion (above K_{crit} in Fig. 1D). ***Nondivisional cell elongation under FtsZ dysregulation)*** Subcritical unbalances in u_h cause the protein field to remain homogenous at subcritical conditions, thus no membrane constriction happens; in vivo, constriction failure by FtsZ dysregulation is companioned by cell elongation under non-divisional growth (at $K \leq K_{crit}$ in Fig. 1D).

Minimal phase-field model of bacterial membrane constriction: Energetics. Within the Canham-Helfrich theory, we write the membrane bending free energy in terms of surface curvature and protein concentration as [1]:

$$H(\phi, u) = \int_V \left(\kappa \Phi_{bend}^2 + A_s V_{adh} + A_f V_{osm} + \sigma_\phi |\nabla\phi|^2\right) dV, \qquad \text{(S1)}$$

where κ is the bending modulus of the membrane, which quantifies the energy required to bend the curvature phase field; it reduces to a generalized harmonic change in the curvature strain [6]:

$$\Phi_{bend}(\phi, u) = -\phi + \phi^3 - \epsilon^2 \nabla^2 \phi + \epsilon(1 - \phi^2)\, C_0(u), \qquad \text{(S2)}$$

where ϕ is the phase field order parameter, which takes the value $\phi = 1$ inside the vesicle, and $\phi = -1$ outside; the position of the neutral surface of the membrane is taken to be where $\phi = 0$, and ϵ is the diffuse width of the membrane (see left panel in Figure SN1 for a schematic). In performing energy minimization, we take the limit $\epsilon \to 0$, which corresponds to the Helfrich functional in the asymptotic limit of sharp interface (see Ref. [7] for details). The surface tension coefficient σ_ϕ is the Lagrange multiplier taken in Eq. S1 in case the total area of the membrane is conserved upon changes in curvature. The FtsZ-dependent field of spontaneous curvature is represented by $C_0(u) = \gamma\, u^2$, where $u(\boldsymbol{r}, t)$ is the local concentration of cytokinetic proteins. The parameter γ is chosen as a spring constant that defines the strength of the molecular membrane curvature stressor, which impart spontaneous curvature in dependence of a harmonic field of protein concentration

at feedback response of local membrane curvature. Let's notice that at the considered level of coarse-graining (see Fig. SN1), the protein field actually represents a number of cytokinetic proteins (driving FtsZ, anchoring proteins FtsA and ZipA, and all the other accessory proteins that constitute the cytokinetic engine [8,9]); for simplicity, we will hereinafter refer to as FtsZ-based cytokinetic engine, or Z rings (see Fig. SN1). Their effective role in our model is twofold: first, imparting membrane curvature (e.g. local constriction but not only); second, generating fields for cohesive interactions between the membrane and the FtsZ-based cytokinetic apparatus (specifically, adhesion and osmotic-like forces, both seeking to concentrate the proteins on the membrane). We describe these ingredients below.

On the one hand, the adhesion potential $V_{adh}(\phi, u)$ in Eq. S1 is given by:

$$V_{adh}(\phi, u) = (\phi^2 - 1)^2\ (u - u_{min})^2\ (u - u_{max})^2 + \sigma_u |\nabla u|^2 \qquad \text{(S3)}$$

This adhesion free energy near the membrane $V_{adh}(\phi, u)$ has strength A_s, and has two fixed points $u = u_{max}$ (maximum concentration of protein per membrane site), and $u = u_{min}$ (minimum concentration of protein); as corresponds to a B-model with two minima. This quartic Landau-Ginzburg model is chosen as a minimal description for a biphasic system in which the protein is expected to segregate between regions of maximal and minimal concentration, u_{max} and u_{min}, respectively. The parameter u_{max} measures the affinity of the protein to adhere to the membrane in the Z ringed regions, whereas u_{min} represents the background protein concentration in the membrane regions unable to undergo constriction (usually taken to be zero). The modulation term $(\phi^2 - 1)^2$ hinders the diffusion of the protein away from the membrane, defined as the locus of points such that $\phi = 1$ (as it minimizes the free energy of the protein when located on the membrane. Here, σ_u is the surface tension of the protein field, which allows the protein concentration to diffuse on the membrane while minimising the area of the boundary between protein-rich regions (i.e. Z rings), and protein-poor regions (incompetent in membrane constriction). Eventually, this constraint produces that only one ring remains (coarsening), although several rings are allowed if relaxed (accounting for possible FtsZ dysregulation). Of course, V_{adh} only contributes when the protein is on the membrane and impedes the local concentration of protein to blow up.

On the other hand, the protein osmotic potential far from the membrane $V_{osm}(\phi, u)$, is given by a coupled potential as:

$$V_{osm}(\phi, u) = \phi^2 \left(u - u_{far}\right)^2 \qquad \text{(S4)}$$

with strength A_f, it considers that the average value of the protein concentration far from the membrane has a fixed point at u_{far}.

The potential V_{osm} penalises the presence of protein in the bulk away from the membrane (recall that the membrane is defined as the locus of points such that $\phi = 0$). By exploiting this phase-transition modelling of contractile membranes, we will focus below on the impact of the relevant constitutional modifications in the onset of the criticality that gives rise to cell constriction.

Constriction force and bending resistance: Effective membrane rigidity. The driving forces for the two diffusional fields are represented by the gradient terms in the rightmost parts of these equations. Within the effective approach of this work, these are, respectively, the bending stress $f_b(\phi, u) \equiv \delta H/\delta\phi \sim K_{eff}\phi$, which represents the effectively linear resistance of the membrane to flexural deformations, and the nonlinear contractile stress $f_c(\phi, u) \equiv \delta H/\delta u \sim A_s u_h^3 + \cdots$ (in the highest order of the FtsZ membrane adhesion term), which represents the membrane deforming forces exerted by competent Z rings leading cell division. Both stresses are composed of membrane elasticity and protein distribution coupled together in a force trade-off which, depending of the constitutional conditions (effective membrane rigidity K_{eff}, and mean FtsZ concentration u_h), gives rise to a systemic mechanical equilibrium either as a stable membrane (nonevolving), or an unstable membrane (leading cell division).

Whereas the FtsZ level u_h is assumed a systemic invariant of our theoretical model [1], however, $K_{eff}[\kappa, A_i, \sigma_i, C_0(u)]$ is actually composed of more fundamental mechanical properties, these are; the putative bending rigidity (κ), the lateral tensions (σ_ϕ and σ_u), and the FtsZ-dependent spontaneous curvature $C_0(u)$, which altogether contribute to the effective membrane stiffness that opposes to the contractile action of the protein field. For an expanded discussion and further details on the relevant parametric space, we refer the reader to the previous paper in Ref. [1]. Note that in our model K_{eff} is allowed to depend on the protein concentration through of $C_0(u)$, i.e., the protein field created by the cytokinetic proteins FtsZ, FtsA and the other components of the construction apparatus; altogether, they are also able to modify the effective membrane rigidity by imparting curvature. Intuitively, the local increase in the concentration of protein increases the spontaneous curvature; such an effect is induced by the harmonic dependence of the local spontaneous curvature as $C_0 = \gamma u^2$ [1]. Therefore, regarding the bending strain field defined in Eq. S2, positive spontaneous curvature contributes to effectively rigidify the membrane (K_{eff} increasing with $\gamma > 0$), whereas negative spontaneous curvature causes effective softening (K_{eff} decreasing with $\gamma < 0$).

Membrane dynamics. The dynamical equations of both fields are taken to follow the Canh-Hilliard model, since u and ϕ are transported by diffusion,

$$\frac{\partial \phi}{\partial t} = D_{\phi} \nabla^2 \left(\frac{\delta H}{\delta \phi}\right) \quad \text{(S5)}$$

$$\frac{\partial u}{\partial t} = D_u \nabla^2 \left(\frac{\delta H}{\delta u}\right) \quad \text{(S6)}$$

where D_{ϕ} and D_u are the corresponding diffusion coefficients, which give the two characteristic time scales of the system; the membrane diffusivity and the protein diffusivity. We assume the protein dynamics to be slower than curvature changes; i.e. the evolution of the protein field is dynamically subsidiary to the shape changes, which stablishes a remodelling kinetics driven upon bending rigidity. For an expanded discussion and further details on the critical membrane dynamics, we refer the reader to the previous paper in Ref. [1]. The dynamical approach below will be limited to the analysis of the unstable membrane modes of protein membrane concentration able to lead constriction as a local coarsening of cytokinetic protein as competent Z rings.

Linear stability analysis: rate of unstable mode leading constriction. Because the total concentration of the driving protein FtsZ is a conserved quantity (u_h), a dispersion relation describing its membrane dynamics $\omega(q)$ could be obtained through a linear stability analysis of the systemic Eq. S6 as subsidiary to Eq. S5. This analysis is performed below, revealing the appearance of a non-linear instability (when $\omega(q) > 0$), which depends crucially on the constitutional parameters κ and u_h. In order to analyse the stability of a homogeneous distribution of protein on the cell membrane, one can study the effect of small perturbations on this state. In particular, we consider that perturbations take the form of plane waves: $u = u_h + u_0 e^{iqx+\omega t}$ and $\phi = \phi_0 e^{iqx+\omega t}$ varying around the membrane (placed at $\phi = 0$) and u_h, which is the concentration of protein, initially homogeneously distributed on the membrane. We further assume fluctuations in the domain of low amplitudes $\phi_0 \ll 1$ and $u_0/u_h \ll 1$. Substituting these solutions in the linearized Canh-Hilliard version of the transport equations (Eqs. S5 and S6), we obtain two different real roots for the dispersion relation $\omega(q)$. One of them is shown to exhibit a region of values of wavevectors q such that $Re[\omega(q)] > 0$, which indicates membrane instability; no oscillatory roots are considered. The variable $Re[\omega(q)] > 0$ represents the rate at which the unstable mode grows exponentially, being thus understood as a membrane relaxation rate for the critically phase-separating protein field undergoing spinodal decomposition into Z rings; this process works as the kinetic bottleneck that determines the effective rate for cell division. The Figure 1C in the main text shows both the stable ($\omega(q) < 0$) and unstable ($\omega(q) > 0$) branches for three different values of membrane rigidity around a value κ_0 representing normal constrictional conditions. Because membrane constriction occurs for an unstable mode of wavevector $q \leq q_{max}$, we stablish $(\partial\omega/\partial q)_{q=q_{max}} = 0$ as the

dynamic condition for critical conditions leading the bacteria for divisional pinching [1]. Consequently, the constriction rate is expected at the maximal rate compatible with this wavevector, this is $\omega_{max}(\kappa) = \omega(q = q_{max})$. Therefore, we stablish the critical divisional rate as $\omega_{max}(\kappa)$, which is the effective growth rate considered in our further analysis of the proliferation dynamics (See Supplementary Note N5).

As a main conclusion in view of Figure 1C, the higher κ (membrane stiffening), the slower the constriction rate till reaching complete dynamic hindering at $\omega_{max}(\kappa_{crit}) = 0$. Contrarily, the lower κ (membrane softening), the faster. By means of this linear analysis, we have also concluded that the spontaneous curvature, which couples the changes in the concentration of protein with the perturbation of the shape of the membrane, regulates dynamics at the onset of the instability; the higher the negative curvature, the faster. Noticeably, the dynamic condition $\omega_{max}(\kappa)$ arises exclusively from a trade-off between membrane rigidity and FtsZ concentration; no choices of membrane dimensions or membrane boundary conditions are involved in these calculations. Consequently, the kinetic parameter $\omega_{max}(\kappa)$ can be considered as the relevant divisional rate for given κ and u_h (see Fig. 1D), independently of a given choice for cell dimensions. Therefore, the dataset in Figure 1D represents the relevant parametrization to describe divisional rates in terms of generalized membrane rigidity and FtsZ content as performed in the analysis of heterogenous population dynamics detailed in the Supplementary Note N5.

Supplementary Note N2. Rheological study with model E. coli lipid membranes: Bilayer GUVs and Langmuir monolayers.

Experimental rationale. As a complementary study on the mechanical impact of molecular inclusion agents in the lipid membranes, we explored in vitro, the rheological behavior of model E. coli membranes in the presence of variable concentrations of pentanol and dodecylamine (see Fig. SN2-1 below). To determine the effect of the putative elastoactive agents, we considered membranes based on the E. coli polar lipid extract (PLE), which is a natural product obtained from the inner lipid membrane of these bacteria (commercially available at high purity). The composition of this E. coli PLE is phosphatidylethanolamine (PE: 67.0 wt%), phosphatidylglycerol (PG: 23.2 wt%) and cardiolipin (CL: 9.8 wt%). Both bilayers and monolayers made of E. coli PLE lipids were prepared mixed with (or in the presence of) the membrane additives as follows.

Fluctuation spectroscopy in giant unilamellar vesicles (GUVs): bilayer bending modulus. We prepared giant unilamellar vesicles (GUVs) by electroswelling [10]. From a solution of PLE dissolved in chloroform at a

concentration of 5.5 mg/ml, small drops of 15 µl was placed on an ITO plate. Once the chloroform was evaporated, the space between the ITO plates was filled with a solution of sucrose at 220 mM. The chamber arrangement was sealed with a nonconductive wax to prevent leaking and evaporation. An AC voltage of 1.7 V at 500 Hz was set for 16 hours. Then, we took advantage of the shape fluctuation method [11], which allowed for bending modulus measurements in GUVs of E. coli PLE prepared with variable amounts of elastoactive membrane modifiers (as described in Methods). The membrane fluctuations were tracked with an inverted microscope Nikon Eclipse Ti at phase-contrast mode, using an oil immersion objective (Plan Achromat 100x, NA 1.45), and an ultrafast CMOS Camera (Photron FastCAM SA3) under cold white LED illumination. Images were stored on a computer and analyzed with a custom software running the segmentation algorithm on parallel in GPU [11]. Once the equatorial cell contour is segmented $\boldsymbol{h}(\boldsymbol{x})$, the fluctuation spectra is computed as in Pecréaux et al. [12]:

$$\langle |u_q|^2 \rangle = \frac{\pi \langle R \rangle^3}{2} \left[\langle |c_q|^2 \rangle - \langle |c_q| \rangle^2 \right], \tag{S7}$$

from the Fourier transform

$$c_q = \oint_0^{2\pi} e^{-iqx} h(x)\, dx \tag{S8}$$

as digitally computed by FFT for the azimuthal wave vectors $q_l = l/\langle R \rangle$ ($l = 2, 3, \ldots$), corresponding to the average radius $\langle R \rangle$ determined in the in the equatorial *x*-plane [13,14]. The quantity u_q represents the amplitude contribution from mode q to the normal membrane displacements with respect to its mean position at $\langle R \rangle$. This equatorial spectrum is related to the Helfrich bending free energy through the membrane tension (σ), and the bending modulus (κ), as follows [12]:

$$\langle |u_q, y = 0|^2 \rangle = \frac{k_B T}{2\sigma} \left[q^{-1} - \left(\frac{\sigma}{\kappa} + q^2 \right)^{-1/2} \right] \tag{S9}$$

Using this spectroscopic schema with the vesicle shape fluctuations, we studied GUVs made of E. coli PLE doped with the membrane modifiers (pentanol and DDA); the obtained results are shown in Figure SN2-1 below.

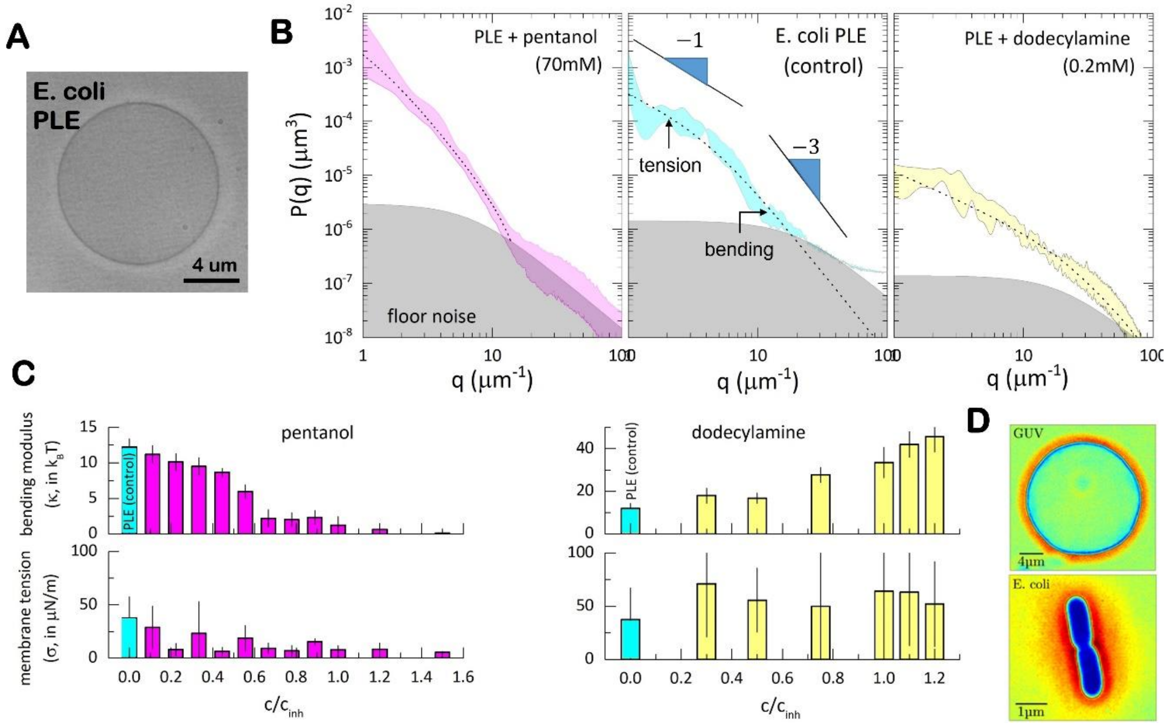

Figure SN2-1. A) Electroformed GUV made of E. coli PLE suspended in LB buffer (in which the membrane modifiers are dissolved). We typically analyzed twenty GUV specimens per case (the raw movies can be provided upon request to the authors). **B)** Experimental fluctuation spectra of GUVs of E. coli PLE (central panel), and those doped with the two elastoactive additives used in this work: pentanol (left panel); DDA (right panel). The Fourier spectra are obtained from the vesicle shape fluctuations observed in the equatorial plane, which are analyzed in terms of Eqs. (SN2-1/2) (data corresponding to two representative concentrations of the doping agents). The dashed region represents the variability band in a population of vesicles (typically N ≈ 20), which determines the standard deviation of the mechanical parameters as obtained from the fits of the experimental spectra of the vesicle shape fluctuations to the Helfrich spectrum in Eq. (S9) (dashed lines). The lower spectral region shadowed in grey corresponds to the instrumental floor noise affecting these experiments (spectrally distributed as a Lorentzian uncertainty due to image pixelization of the fluctuation movies). **C)** Mechanical parameters as obtained from the best fits of the equatorial spectra to Eq. (SN2-3): bending rigidity (top panels); membrane tension (down panel). The bar charts show statistically significant properties expressed in terms of the concentration of additives: pentanol (left); DDA (right), with respect to the control case of bare membranes made of E. coli PLE (bars colored in cyan). The relative concentrations are referred to the inhibitory dose of these antibiotics in wild-type E. coli ($c_{inh} = 90mM$ for pentanol, and $c_{inh} = 0.22mM$ for DDA). **D)** Compared microscopy photoshoot of a typical GUV and an E. coli cell as imaged in the phase-contrast mode for quantitative mechanical analyses by fluctuation spectroscopy.

The above results showed the vesicles made of E. coli PLE lipids (without additive) characterized by a relatively low value of the bending modulus $\kappa_0 = 12 \pm 2\, k_BT$ (average over $N = 20$ vesicles) [10], similarly to vesicles made of unsaturated

phospholipids [15], and of charged phospholipids [16]. The PLE is mainly composed of phosphatidylethanolamine (67%), phosphatidylglycerol (23%) and cardiolipin (10%), which determines the E. coli lipid bilayers with a dominant negative charge. As expected, addition of the elastoactive membrane modifiers resulted into a strong impact in the bending rigidity of the charged PLE bilayer (see Fig. SN2-1C). Particularly, adding dodecylamine-hydrochloride (DDA), at similar concentrations than in the growth inhibition range observed in the bacterial cultures ($\boldsymbol{c < c_{inh}^{(DDA)}} \approx 0.22mM$), caused a significant increase of the bending modulus by a 3-fold factor the value found for the bare PLE lipids. Conversely, adding pentanol caused a very significant decrease down to zero rigidity, with the bending modulus vanishing at concentrations close to the inhibition concentration ($c < c_{inh}^{(C5OH)} \approx 90mM$). Our present results on the altered bending rigidity of modified lipid membranes are compatible with previous, more systematic studies performed with micropipettes [17,18], fluctuation spectroscopy [18,19] and scattering techniques [20,21].

Langmuir monolayers: compression modulus. In order to quantify the compression modulus that determines the lateral rigidity of the lipid membrane, we also prepared Langmuir monolayers of the E. coli PLE. Insoluble monolayers were formed by spreading from chloroform solution (0.5 mg/mL) on an aqueous subphase (LB buffer, Mg2+ 2mM); we poured drops with a Hamilton syringe at the surface area comprised between the two barriers of a computer-controlled Langmuir trough (NIMA, England). In all experiments, 50μL of lipid solution were spread on a buffered subphase containing different amounts of the surface modifiers (pentanol, DDA and others) at specific concentrations determined by their respective growth inhibition concentrations measured in the bacterial culturing experiments. After an awaiting period of solvent evaporation (10 min), the surface pressure-area isotherms ($\pi - A$) were obtained by continuous compression at a constant velocity of 0.1 cm2/s. The lateral compression modulus of the lipid monolayer was defined as:

$$\epsilon = -A(\partial\pi/\partial A)_T, \quad \text{(S10)}$$

which was calculated as a numerical derivative at every surface state defined at isothermal conditions. Measurements were performed from an initial area of 270 cm2 down to 60 cm2, which allows the compression of the monolayer from the gas-like state till collapse. All the area compression experiments reported in this work were performed at 37ºC, with the temperature controlled by recirculating water from an isothermal bath. In order to determine the impact of the additives on the lateral ordering of the lipid membranes, we further studied the compression modulus (ϵ) of the Langmuir monolayers of the E. coli PLE prepared in the presence of variable amounts of the membrane modifiers. The experimental results are plotted below in Figure SN2-2.

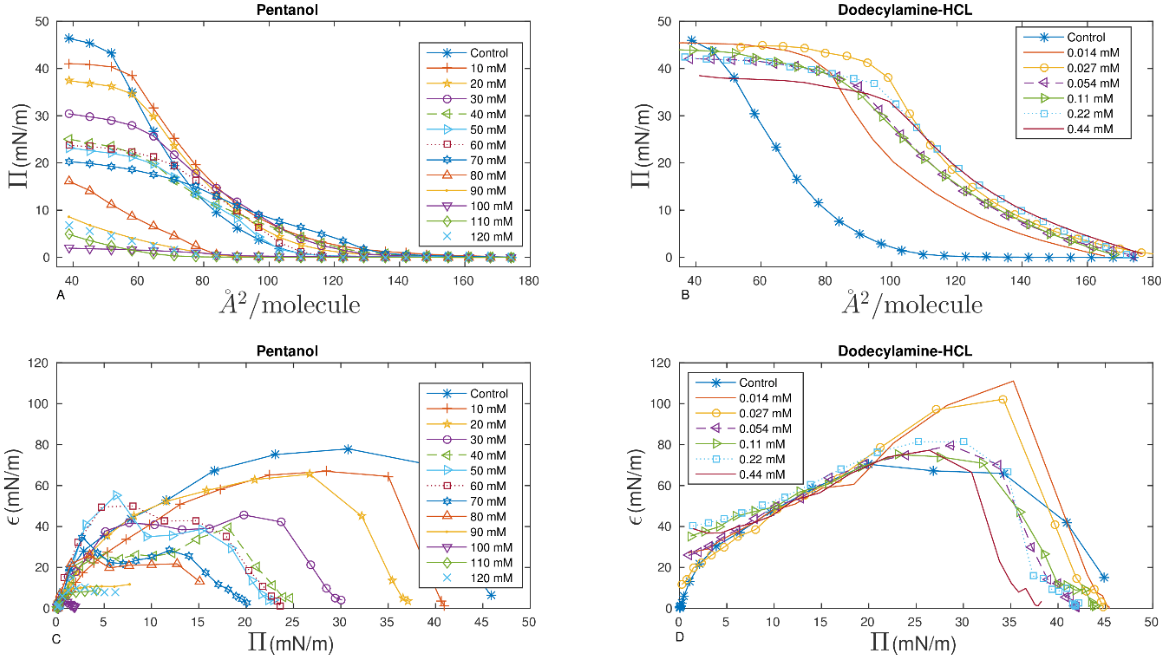

Figure SN2-2. Top panels) Compression isotherms of PLE Langmuir monolayers (top panels): effect of pentanol (left) and DDA (right). Down panels) Pressure dependence of the compression modulus defined as $\epsilon = -A(\partial\pi/\partial A)_T$ (calculated as the numerical derivative of the compression isotherms in the top panels).

From the compression isotherms (see Fig. SN2-2; top panels), under pressure changes at constant area we detected either depressurizing expansion upon pentanol insertion (left), or pressuring compaction upon DDA insertion (right). The quantitative analysis of the compression modulus allowed for a detailed analysis of their impact in the molecular packing (see Fig. SN2-2; down panels). For the bare monolayers of the PLE at the reference state representative for the molecular packing in the bilayer (at the monolayer equivalent surface pressure $\pi_b = 30\,mN/m$) [22], we measured $\epsilon_0 = 80 \pm 10\ mN/m$, a value typical of disordered

lipid membranes in the fluid state [22,23]. For the equivalent bilayer, under weak intermonolayer interactions upon the high strength of the lateral packing forces, the compression modulus is assumed near twice the monolayer value; for the reference state, this is $E_0 \cong 2\epsilon_0 = 160 \pm 20\ mN/m$, in agreement with values measured for vesicles of unsaturated phospholipids by micropipette aspiration [17,22]. Regarding the impact of additives, whereas adding DDA caused only a weak increase of the compression modulus ($E \gtrsim E_0$, by 20% maximum) indicative of a certain ordering effect, the presence of increasing amounts of pentanol in the aqueous phase caused a dramatic decrease of the compression modulus down to vanishing values ($E \to 0$), which revealed the strong disordering character of this short-chain alcohol on the lipid membrane.

The results with the rigidified membranes using DDA were found compatible with previously published data for unsaturated phospholipid monolayers doped with similar fatty substances [22-24]. From those extensive compilations, we found values ranging $\epsilon \approx 80 - 120\, mN/m$ for the rigid monolayers of hydrophobic substances in the liquid condensed state (corresponding to $E \approx 160 - 250\ mN/m$ for the bilayers). However, the small hydrophilic molecules are known to modulate the lipid phase transitions as monolayer expanders that localize near the polar headgroups [22,23]. Similar softening than observed here for PLE lipid monolayers penetrated by pentanol has been reported for phospholipid monolayers in the presence of propofol [25], and magainin [26]. Our mechanical results with the softened membranes were compatible with a systematic report on the structural disordering effect induced by short-chain alcohols on bilayer vesicles made of unsaturated phosphocholines [27].

Molecular mechanics of the modified E. coli membranes: bending rigidity vs. compression modulus. A compared analysis of the dependencies of the transverse bending rigidity (κ), in terms of the longitudinal compression modulus (E), will conduct us to a deeper understanding of the molecular bases of the mechanical effects induced by the membrane modifiers. As an approach from the continuous mechanics of these membranes, Figure SN2-3 shows the compared results as got from the above rheological study.

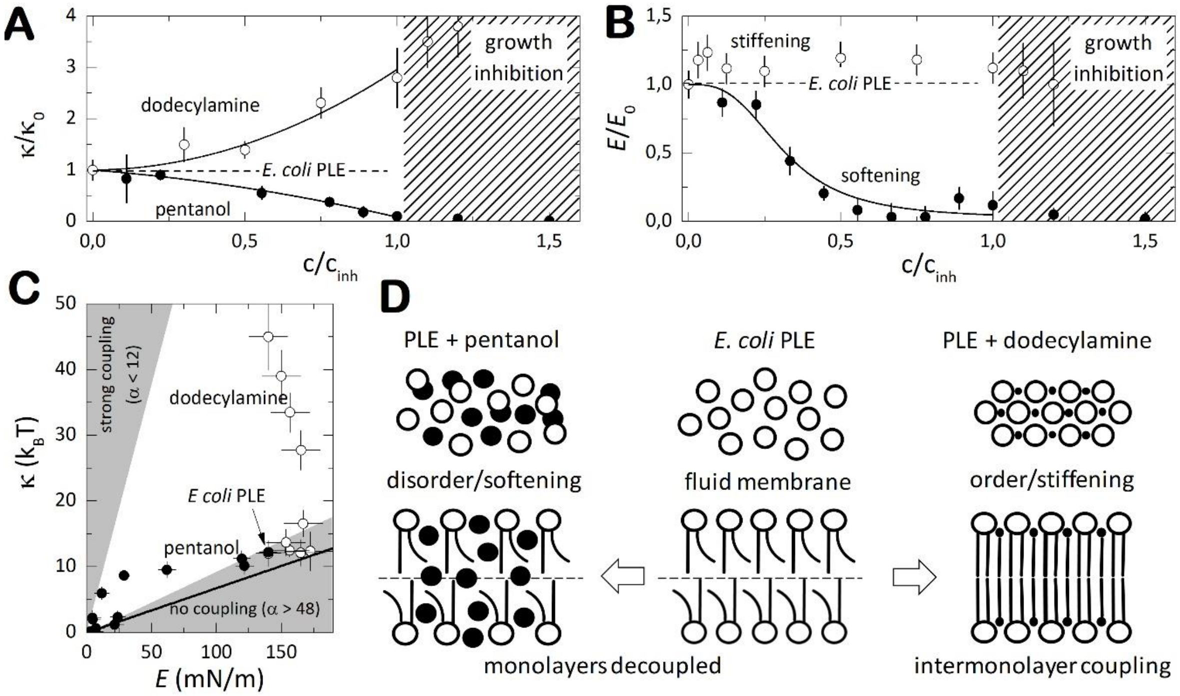

Figure SN2-3. A) Relative mechanical impact of the two elastoactive membrane additives, pentanol, and dodecylamine (DDA), on the bending modulus of giant unilamellar vesicles based on E. coli PLE bilayers (κ/κ_0 with $\kappa_0 = 12\,k_BT$ being the reference value for bare E. coli lipids). Whereas pentanol contributes to decrease κ, addition of dodecylamine conversely makes to increase the value of this quantity. **B)** Relative impact of the two additives on the compression modulus of the monolayer leaflets (E/E_0 with $E_0 = 160\ mN/m$ being the reference value for bare E. coli lipids). **C)** Cross-correlation of the membrane bending rigidities (transverse stiffness) and the lateral compression moduli (in-plane compressibility). **D)** Cartoon for the structural scenario depicted from the mechanical results in **C).** Addition of short-chain surfactants causes structural disorder on the fluid lipid bilayer (left), whereas long-chain fatty substances induce lateral ordering and increase the degree of intermonolayer coupling (right).

Adding pentanol caused a sigmoidal like decrease of the bilayer bending rigidity (see Fig. SN2-3A), with κ decaying from the value corresponding to the PLE down to zero in approaching the inhibitory concentration ($c_{inh}^{(C5OH)} \approx 90mM$); in a similar way, the compression modulus was impacted by this softening alcohol as decreasing $E_{C5OH} < E_0$ (see Fig. SN2-3B). Conversely, DDA was observed with a strong stiffening impact in the bilayer bending rigidity due to the insertion of the hydrophobic fatty chains with a small primary amine head (see Fig. SN2-3A). Despite the global monolayer expansion caused upon fatty amine insertion (see Fig. SN2-2), a weak increase was detected in the compression modulus (at low DDA concentration); this compaction is probably caused by monolayer

condensation at the level of the hydrophobic chains. Further DDA addition does not produce additional condensation, the compression modulus remaining essentially constant at a value only a 10-20% higher than for the bare PLE membranes; this is $E_{DDA} \approx 1.5E_0$ (see Fig. SN2-3B). These combined results showed a subtle dichotomy: whereas the hydrophobic membrane modifier (DDA) mainly causes a strong impact on the transverse resilience of the bilayer companioned by a weak lateral compaction of the monolayers (measured as a weak increase of the compression modulus followed by a strong increase of the bending rigidity), the hydrophilic additive (pentanol) elicits a strong disturbance on the lateral packing that leads to monolayer expanding followed by a softening of the bilayer (proportionally quantified as a decrease of the bending rigidity). Such a dichotomy stems on the different nature of the mechanical interplay between lateral and transverse interactions in the two cases. This intercoupled compression/bending mechanics deserves an insightful discussion that will help to enlighten the adequate biophysical mechanism required for a maximal antibiotic action of the elastoactive membrane modifiers.

Mechanical interplay between bending and compression: Intermonolayer coupling. The lipid bilayer of biological membranes is composed of two monolayer sheets weakly held together by normal stresses giving rise to intermonolayer coupling [28,29], which mainly underlies tail-to-tail interdigitation interactions and rapid transbilayer transport, among other important features [30]. Depending on the degree of intermonolayer coupling the monolayers are restrained to move relative to one another, thus causing an additional constraint within the composite bilayer structure that gives rise to an additional restoring force (see Fig. SN2-3D). In the simplest description, such energetic interplay considers a coupled oscillator between transverse bending strain and subsequent lateral dilations originated in the two monolayers [28]. Such coupling results in a linear relation between the bending modulus and the compression modulus of the bilayer; as seminally discussed by Helfrich [28], and later modified by Yeung and Evans to account for the effective size of the hydrophobic core of the bilayer [29], the constitutional relationships holds:

$$\kappa(E,d) = \frac{(d-d_0)^2}{\alpha}E \qquad \text{(S11)}$$

where d is the thickness of the monolayer; the difference $d - d_0$ accounts for the effective hydrophobic thickness; α is the intermonolayer coupling parameter, which takes variable values between two extreme cases: a) Ideally coupled bilayer model, with $\alpha = 12$ corresponding to complete coupling between the two monolayers (describing two perfectly stuck monolayers in a static structural scenario, or equivalently, sticking friction in a dynamic scenario). b) Two-monolayers uncoupled model, with $\alpha = 48$ corresponding to a composite bilayer

where the two monolayers freely slide past each other (equivalent to consider sliding frictional conditions between the monolayers in a dynamic scenario). The brush-like model accounts for intermediate coupling scenarios ($12 < \alpha < 48$), where the two monolayers couple at a variable strength depending on their degree of mutual interaction [31].

In view of this intermonolayer coupling theory, which has been extensively verified in a variety of systems [15,19,21,29-31,32], we revisited the experimental results in Fig. SN2-3A/B, and then plotted in Figure SN2-3C the $\kappa - E$ diagram showing the different degree of intermonolayer coupling present in the PLE-based systems here reported with the membrane modifiers (assumed constant membrane thickness). In the case of pentanol, the progressive addition of the short-chain alcohol causes a significant decrease of the lateral compression modulus only followed by a slightly decrease of the bending rigidity. This mechanical behaviour is compatible with an uncoupled intermonolayer scenario ($\alpha = 48$), where the short-chain amphiphilic modifier disrupts the lipid packing in the monolayers at the level of polar heads, but with a weak impact in sticking the monolayers at the bilayer midplane. Similar coupling behaviour is depicted by propofol, a hydrophilic anaesthetic with a molecular structure and a membrane mechanical action occurred through a significant expansion of the constituting monolayers [33]; comparable to the fluidization effect of short-chain amphiphilic alcohols in lipid bilayers [25]. Contrarily, addition of the long-chain DDA caused strong increase of the bending stiffness at moderate in-plane condensation of the constituting monolayers (see Fig. SN2-3A/B), a fact compatible with a strong coupling scenario ($\alpha = 12$; see Fig. SN2-3C), where insertion of the long-tails of the fatty amine elicits additional tail-to-tail structural mismatches at the level of the intermonolayer midplane. Similar strong coupling was depicted by caffeine, a small hydrophobic molecule with a planar moiety able to significantly condense the constituting monolayers [24,34], consequently reducing membrane fluidity [35]; like the condensing effect of cholesterol in eukaryote membranes [36]. The insertion of small amounts of caffeine, which can effectively diffuse between the two monolayers, elicits a global condensation of the bilayer that translates as an increase of the bending stiffness (see Fig. SN2-3C), just like cholesterol does in eukaryote membranes [37]. To conclude from the practical standpoint of this work, even limited monolayer condensation elicited by small amounts of hydrophobic modifiers is expected to cause strong bending rigidification in the bilayer.

Our rheological results have linked bilayer flexibility (transverse bending) with the structural deformability (lateral compressibility) of the constituent monolayers. Two dissimilar structural scenarios have been depicted for every class of additive (see cartoon in Fig. SN2-3D). On the one hand, short-chained pentanol molecules elicit a progressive bending softening on the E. coli lipid membrane, caused by lateral

disordering and intermonolayer decoupling, both induced by a global disruption of the bilayer structure [27]. On the other hand, the fatty chains of DDA coherently insert the lipid monolayers [24], resulting in global ordering effects that cause a very significant bending stiffening of the E. coli membrane, due in part to lateral ordering in the monolayers, but mainly to a strong increase of the degree of intermonolayer coupling as here revealed from the combined data in Fig. SN2-3C.

As a plausible in vivo implication of these conclusions (achieved in vitro), we expect any structural modification of the bacterial bilayers with a potential impact on the mechanical parameters that determine the effective rigidity of the real biomembranes (in vivo, after metabolic processing; see Fig. SN1). Particularly, lipid bilayer compaction is expected to contribute higher bending stiffness κ and lateral tension σ_{ϕ}, with a direct impact at increasing K_{eff} (as a lipid reinforce to the rigidity imposed by the PG wall). Conversely, lipid decompaction is expected to cause lateral distension and bending softening (decreasing $\sigma_{\phi}$and κ, respectively), with a direct impact at decreasing K_{eff} (at the bare value fixed by the PG layer).

Supplementary Note N3. Synchronized bacterial culturing.

Experimental: synchronized cultures. We used E. coli strain MG1655, which was cultured in order to obtain bacterial growth curves. All cultures were prepared in Luria Bertani medium broth at a constant temperature of 37 ºC. Starting from an overnight culture of 2 mL, we standardize the state of the inoculum through a series of 4 consecutive dilutions. The first dilution starts with a relation of 1:100 (20 uL of the overnight culture in 20 mL of medium). When this culture reached an absorbance of 0.3 (at 600 nm), it starts a process of 3 consecutive dilutions with a relation of 2:1 (10 mL the inoculum in 20 mL of medium), every dilution starts when the previous inoculum reached an absorbance of 0.3 (at 600 nm). From the final inoculum 200 uL were taken and 2.4 mL of LB medium was added at different concentrations of the elastoactive agents pentanol and dodecylamine-hydrochloride (DDA) previously dissolved in the LB medium. From every solution, 200 uL were taken and poured into a flat-bottomed 96-well microplate. All the samples in the microplate were not sealed in order to allow for adequate oxygenation of the bacterial cultures. Growth curves were obtained using a spectrophotometer Multiskan GO Thermo Scientific at 600 nM and settled to a constant temperature of 37 ºC with continuous shaking between consecutive measurements. Each culture was replicated five times in the same microplate, and each experimental condition was repeated by five times in independent experiments. The uncertainty in the averaged values of the experimental parameters was obtained as a standard deviation measured over the $N = 25$ replica considered at every experimental condition. The standard deviation of the

resulting averages is lower than 1% (typically smaller than the point size in the turbidity plots).

Analytics: logistic growth model; homogenous case. The experimental growth curves were quantitatively fitted to a general logistic growth-model defined for the observed absorbance (an optical density OD) as a function of time (t) as:

$$Q(t) = Q_0 + \frac{Q_\infty - Q_0}{1 + exp\left[-G_{eff}(t - t_0)\right]} \qquad \text{(S12)}$$

where the fitting parameters are the effective growth rate (G_{eff}), as the slope of the curve in the exponential phase, the lag time (t_0), and the maximum absorbance (Q_∞), as referred to the initial value (Q_0).

At steady-state in the exponential phase, homogenous populations of identical specimens grow non-delayed (log phase $t_0 \approx 0$); thus $G_{eff}(t - t_0) \approx G_{eff}^{(homo)} t \gg 1$, so that the generalized kinetic equation reduces to the simpler case $Q(t) \approx Q_\infty exp\left[G_{eff}^{(homo)} t\right]$, which represents the bare exponential growth in Eq. (1) of the main text (for $Q_0 \approx 0$). In this homogenous case, the effective growth rate measures the instantaneous velocity of exponential proliferation of the bacterial colony; at the log phase this is $G_{eff} = (1/N)(dN/dt)$, with N being the number of specimens. During steady-state exponential growth of homogenous populations, regulated cell traits such as cell size, DNA and RNA content, FtsZ level, etc. per cell remain constant in time, which justifies the homogeneous logistic growth characterized by a doubling rate directly determined by the division rate of the single individuals, i.e. $G_{eff}^{(homo)} = ln2/\tau_p$, with τ_p being the doubling time defined for the exponential growth (see Eq. 1 in the main text). In the presence of environmental disturbances (such as elastoactive treatment, among others), only if the cell division process is altered without perturbing the DNA cycle and all other biosynthesis, both individual cell and population still grow exponentially with the same growth rate as given by a constant doubling time [38].

Heterogenous case; delayed proliferation dynamics. In exponential culture of E. coli growing with additives, changes in the division rate are not necessarily the same as changes in the growth rate. The reason is that the elemental division rate is controlled by the DNA replication time [38,39], whereas the growth rate is determined by the ribosome content active in gene expression as the key factor for cell growth [40,41]. Furthermore, there is extensive evidence that the growth rate is coordinated with the cell size [42]. In Gram-negative bacilli, E. coli among them, the average cell size increases with the nutrient imposed growth rate [43]. Indeed, alterations in cell division rates are classically known with effects on the cell size [38,39], a phenomenological observation recently revisited as a predictable fact

from more basic principles [44]. Therefore, the changes in growth rate elicited by the elastoactive additives cannot be simply interpreted as changes in division rate and require further cytometric evidence revealing heterogeneities in cell size that could determine a structured proliferation kinetics (as modelled form first principles in Supplementary Note N5). In more phenomenological terms, we will consider the generalized model of bacterial growth as postulated by Hills and Wright (HW) for heterogeneous populations with a structured kinetics determined by the cell biomass of the different individuals growing above a minimal cell size corresponding to the slowest viable subpopulation [45]. The HW-model derivates the same generalized kinetic equation as in Eq. S12, describing heterogenous colony proliferation with an effective growth rate $G_{eff}^{(het)}$, as given by the apparent slope of the log phase. The lag time t_0 accounts for the divisional delay imposed by the biggest specimens taking longer times to divide after elongation.

Supplementary Note N4. Single cell tracking of membrane fluctuations in spherocylindrical bacteria: Mechanical maps.

After the standardization process described above, we took 200 uL of the inoculum and 2.4 mL of LB medium was added at different concentrations of pentanol and DDA previously dissolved in the medium. From every solution it was taken a small drop and poured in a hot drop of LB medium with agarose (2% volume) to mildly adhere the bacteria on the microscope slide thus hindering them for translational and rotational mobility. Agarose slide treatment: A drop of 5 uL of agarose (warmed) was deposited onto the coverslide and let it cool. Over the mattress of agarose, a single drop of 10 uL containing cells in culture medium was placed letting the cells in contact with agarose mildly adhere. Measurements of the vibrational fluctuation of the membrane of E. coli were taken with the experimental array described early with an average of 10,000 images taken at 2,000 FPS.

Image processing algorithm and cell contour segmentation at normal membrane directions. The fluctuations of the membrane along the normal directions were obtained through the analysis of the white halo visualized at the membrane emplacement with phase-contrast microscopy. The cell contours optically focused at the equatorial plane were digitally segmented using a custom-made algorithm [11], which combines accurate positioning of the membrane contour with respect to centroid [46], and optimal contour imaging with respect to background noise [47]. To track membrane fluctuations in sphero-cylindrical contours, we specifically adapted this algorithm to determine membrane positions along the normal directions. We modified the algorithm of contour segmentation previously developed for spherical vesicle geometries in Ref. [11]. First, for each

spherocylindric contour frame, we run the previous algorithm to detect its centroid starting from an arbitrary point of the contour in the vertical direction. In order to get an initial contour of the bacteria, we performed 2048 radial scans at a constant angle difference between rays (see Figure SN4-1A).

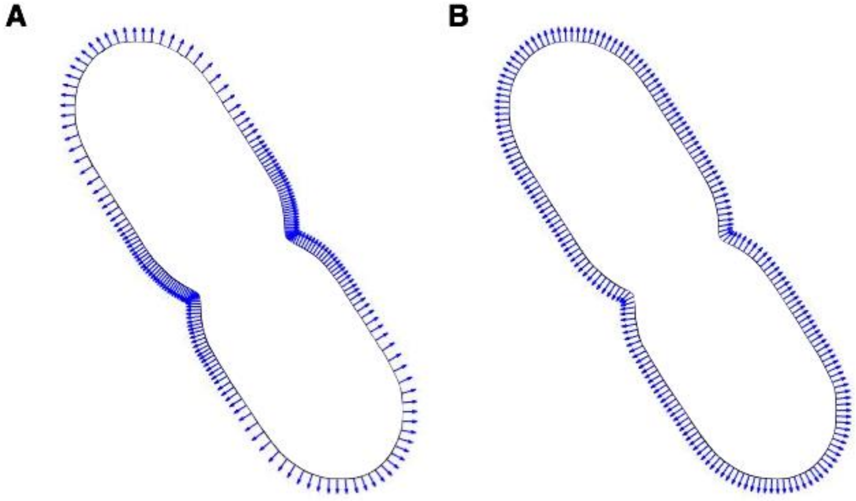

Figure SN4-1. Schematics showing the direction of the normal membrane vectors used for the detection of the membrane fluctuations of bacteria. **A) Radial fluctuation framework.** Former radial frame obtained through the spherical scan of the cell contour using the algorithm in Ref. [11]. A spurious concentration of segmented sectors occurs in approaching the curvature discontinuity in the division site. This caused apparently abnormal density fluctuations leading to interpretation artifacts. **B) Normal framework.** Correction for normal frame directions at each membrane location using a cubic spline interpolation. The normal direction was calculated using the position of the neighbors for every ray to calculate the normal vector.

To correct the fluctuations from the initial radial distribution to the normal membrane directions, thus avoiding artifacts due to the intersection of the rays in the concave sections of the contour, the equidistant position of the 128 points was recalculated using interpolation from the initial contour with a cubic spline approximation (see Figure SN4-1B). For each point, it was calculated a normal vector using the position of the closest twenty neighbors as best matched to the spline interpolator. The centroid and normal vectors were recalculated in an iterative way if the centroid was displaced beyond a threshold value. This process is repeated for the whole set of images. A minimal tolerance of a 0.1% was chosen as a criterium for convergence. The position displacements of the membrane $h(x,t)$ are calculated along these normal directions. From the normalized histograms of these membrane height displacements at each membrane emplacement $x = x_i, h(x_i,t)$ for $i = 1, \dots 128$, we obtained the ensemble-averaged

PDFs of the membrane fluctuations (as determined by the time averages $\langle\ \rangle_t$ calculated over the fluctuation series $h(x_i,t)$).

Mechanical maps of fluctuations. The PDF is used to calculate the three first consecutive moments of the displacement distributions at each membrane emplacement, i.e. $PDF\{h(x_i,t)\}$; these moments are: first, the standard deviation (SD); second, the skewness (S); and third, the kurtosis (K). The effective membrane rigidity K can be calculated from the local variance, this is $\sigma_h^2 \equiv \langle h^2\rangle_t - \langle h\rangle_t^2$; considering the whole cell contour, we inferred $K \simeq Ak_BT/\Sigma_h^2$ as a spatial average calculated from $\Sigma_h^2 \equiv \langle\sigma_h^2\rangle_x$. The other two momenta (S and K) allowed for the analysis of possible statistical deviations from Gaussianity due to active (nonthermal) contributions to the membrane fluctuations [10]. As a proof of validation of our segmentation method to track membrane fluctuations in spherocylindrical contours, in Figure SN4-2 we tested the modified algorithm in synthetic bacterial profiles mimetic of dividing E. coli cells. The synthetized fluctuations were generated as a white noise with a random distribution along the spatial cell profile. We analyzed their statistical traits from the PDFs, which were locally determined as spatial membrane maps at each point in the cell contours (see Fig. SN4-2 below). The method was validated with a high performance and a high accuracy in determining the normal fluctuations at subpixel resolution, even at the low signal-to-noise conditions tested in the artificial setting of the synthetic profiles (see caption for details).

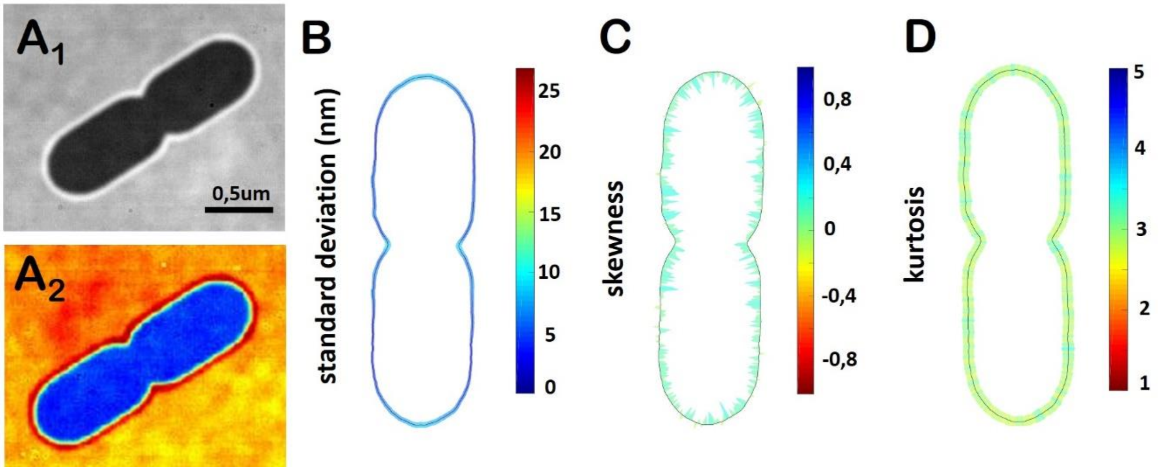

Figure SN4-2. Proof of validation of the membrane fluctuation tracking algorithm in spherocylindrical bacteria. A) We created synthetic profiles of Escherichia coli undergoing equatorial constriction. The artificial images were synthetized with a structure and size like the real thing is observed under the phase contrast microscope including a noisy background (panel A_1). The synthetic cell profiles were locally endowed with a fluctuating membrane aspect as given by averaged coordinates affected by a white noise with a Gaussian distribution of unity standard

deviation. By using the high-precision algorithm in Ref. [11], the cell profile was properly segmented at the central position of the contrast halo (as revealed in the false color image of panel A_2). Membrane fluctuations were tracked in the normal directions with the specific algorithm developed in this work for spherocylindrical cell contours (see above for details). The statistical characteristics of the local PDFs were calculated and mapped along the spatial coordinate: **B)** First moment: standard deviation; **C)** second moment: skewness; **D)** third moment: kurtosis. These fluctuation traits were properly mapped in accordance with the synthetic characteristics imposed to the Gaussian noise simulation (SD: $\sigma_h = 10nm$ $(\pm 20\%)$; skewness: $S = 0;$ kurtosis: $K = 3$).

For the sake of example in the biological setting, Figure SN4-3 shows the algorithm at work with the normal membrane fluctuations of a real E. coli specimen. In those cases, cell contour segmentation was also efficient to resolve the membrane fluctuations at enough tracking performance. The method was further exploited with living E. coli cells treated with elastoactive additives (see Figure 4 in the main text, and Figures S5-S6 in these Supplementary Materials).

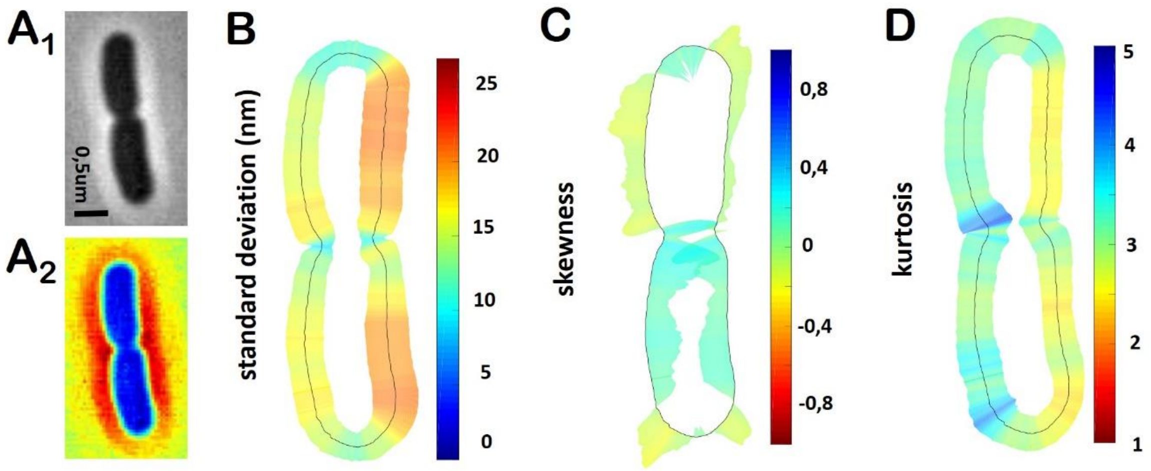

Figure SN4-3. Tracking normal membrane fluctuations in E. coli bacteria. A) Phase-contrast micrograph at 100x magnification (as captured by an oil immersion objective Nikon Plan Apo 100x): Direct black & white image (panel A_1); digitalized in false color (panel A_2). The cell contour was segmented using the algorithm in Ref. [5], and the membrane fluctuations tracked in the normal direction using the method developed in this work (see above for details). **B)** Cell contour segmentation; **C)** Spatial amplitude map of the normal membrane fluctuations (standard deviation, or first PDF moment); **D)** Skewness (second PDF moment); **E)** Kurtosis (third PDF moment).

Supplementary Note N5. Heterogenous population dynamics.

We have developed a model of bacterial proliferation in which cells are assumed to be structured by the content of FtsZ within their membranes. Let us consider $n(t,u)$, i.e. the concentration of bacteria at time t with FtsZ concentration $u(t)$; the time evolution is given by the balance equation:

$$\frac{\partial n}{\partial t} + \frac{du}{dt}\frac{\partial n}{\partial u} = [b(u) - \mu]\, n(t,u), \qquad \text{(S13)}$$

which is subjected to the boundary condition:

$$n\left(t, \frac{u}{2}\right) = 2\alpha\, b(u)\, n(t,u), \qquad \text{(S14)}$$

established as a natural doubling characteristic for cell division prescribed in the population under equitable FtsZ-partitioning in the dividing individuals (with α being the fraction of successful cell division).

The (total) rate of change in the concentration of bacteria occurs due to birth, at a protein-dependent rate $b(u)$, and death at a constant rate μ. The quantity $b(u)$, which is the FtsZ-dependent birth rate, is defined as:

$$b(u) = \tau_p^{-1} H(u - u_{crit}). \qquad \text{(S15)}$$

The definition in Eq. (S15) stablishes that beyond u_{crit}, cells divide after a doubling time τ_p, which is (in principle) assumed to be a natural constant. According to this model, cell do not divide until their FtsZ concentration reaches the instability threshold u_{crit} prescribed for cell constriction by the membrane phase-field theory in the Supplementary Note N1. Regarding the definition of Eq. (S15) in view of the boundary condition where it intervenes (Eq. (S14), the dimensionless parameter $\alpha(u)$ is introduced as a tuneable rate that could depend on the FtsZ level ($\alpha \leq 1$), which reflects the biological factuality for possible specimens able to grow without dividing. This can be observed under appropriate experimental conditions in anomalously growing E. coli filaments with FtsZ expressed severalfold above normal levels [48], or the filamentous polynucleated specimens here obtained under treatment with pentanol (see Fig. 3 in the main text), for instance. In these cases, the fraction of successful cell division upon reaching the critical FtsZ concentration is not a hundred per cent of the apt cells.

Close to the membrane instability assumed in the phase-field model (leading constrictional cell division), we have that $u = u_h + u_0 e^{iqx + \omega_{max}(q)t}$, so that:

$$\frac{du}{dt} = \omega_{max}(\kappa)\, u(t). \qquad \text{(S16)}$$

Therefore, we propose a separable form for the solution of Eqs. (S13) and (S14) as $n(t;u) = T(t)F(u)$. Upon substitution of this separable solution, for FtsZ concentrations above a reference level u_0, we obtain:

$$n(t;u) = n_0 e^{\lambda t} exp\left(-\frac{\lambda+\tau_p^{-1}}{\omega_{max}(\kappa)} log\frac{u}{u_0}\right) \quad \text{(S17)}$$

where λ is the associated eigenvalue, which is obtained by replacing Eq. (S17) into the boundary condition in Eq. (S14):

$$\lambda(u;\kappa) = \tau_p^{-1} + \frac{\omega_{max}(\kappa) log(2\tau_p^{-1})}{log(2\alpha)} \quad \text{(S18)}$$

Integrating by the different contents of FtsZ, this separable solution allows us to derive the dynamics for the total population of bacteria as:

$$N(t) = \int_0^{\infty} n(t,u)du, \quad \text{(S19)}$$

which is obtained in differential form as:

$$\frac{dN}{dt} = G_{eff}N, \quad \text{(S20)}$$

where G_{eff} is the effective growth rate as given in function of the FtsZ content and the bending membrane rigidity by $G_{eff}(u;\kappa) = \lambda - \mu$.

Rigidity dependence of the proliferation rates: Effective growth rate. Because we predict $G_{eff} \sim \lambda \sim \omega_{max}(\kappa)$, once the proliferation kinetics is identified as an exponential growth in view of Eq. (S20), from the results in Fig. 5B we posit that, for $G_{eff} > 0$, the growth rate satisfies the following scaling behaviour $G_{eff} \sim (\kappa_{crit} - \kappa)^{\beta}$, where κ_{crit} is the critical value of the bending rigidity above which the instability giving rise to the constriction of the membrane does not occur. To obtain the value of the exponent, we get close to the critical curve and take the difference with respect the corresponding critical value. By analysing the behaviour of $\omega_{max}(\kappa)$ close to the critical value as a power-law, we find that the exponent $\beta \cong 2$ (see Fig. 5B). In this work, we take advantage of this constitutional relationship as a physical connection between the effective proliferation rates observed in the E. coli cultures modified by elastoactive agents and the underlying membrane rigidity in the cell individuals. Such an automatism is not dependent of the biochemical characteristics, thus being potentially universal among different physiological status and organisms. This is a main feature emerged by our results with E. coli under elastoactive treatment, which is enlightened in Figure 5D.

Proliferation delay by deviating optimal FtsZ levels: Lag times. Our model is consistent with a heterogeneous population stratified by levels of FtsZ in the cell individuals; looking at Eq. (S17) compared with the delayed kinetics of the Hills-Wright (HW) model of heterogeneous populations [49] (as described by the

phenomenological law in Eq. 1 of the main text), the cell density in each subpopulation holds a Poisson distribution on the FtsZ concentration as $n(u) \sim n_0 \, exp(-G_{eff} t_0)$, which actually represents a delay term with an exponential lag argument expressed by:

$$G_{eff} t_0 = \frac{\lambda + \tau_p^{-1}}{\omega_{max}} log \frac{u}{u_0}. \qquad \text{(S20)}$$

Because $G_{eff} \sim \tau_p^{-1}$, considering the eigenvalue in Eq. (S18), one can easily deduce a direct connection between the kinetic delay and the FtsZ content through the proportionality relationship $t_0 \sim log(u/u_0)/log(2\alpha)$. Consequently, a zero-lag time is defined for the reference state with an FtsZ concentration regulated at the physiological level for the unmodified wild-type organism, i.e. $t_0 = 0$ at $u = u_0$. However, nonzero lag times are expected with varying FtsZ with respect to normal levels; such u –dependent kinetic delay can be due either to a direct logarithmic increase of the integrating factor for Eq. S17 (i.e. $t_0 \sim log(u/u_0) > 0$ for $u > u_0$), or to an indirect decrease of the fraction of successful cell division with varying FtsZ (i.e. $t_0 \sim 1/log(2\alpha) > 0$ for $\alpha(u) < 1$ at $u \neq u_0$). For the considered E. coli systems, this connection was determined in Figure 5C, which plots the experimental values of the lag argument observed in the bacterial cultures in terms of the rigidity modulus measured in the membrane models. We observed the nontrivial decrease for Gt_0 in approaching the reference state, which assigns the untreated cells proliferating at the onset of criticality, i.e. at $\kappa \approx \kappa_0 < \kappa_{crit}$.

Supplementary Figure S1. E. coli cultures treated with dodecylamine-hydrochloride (DDA).

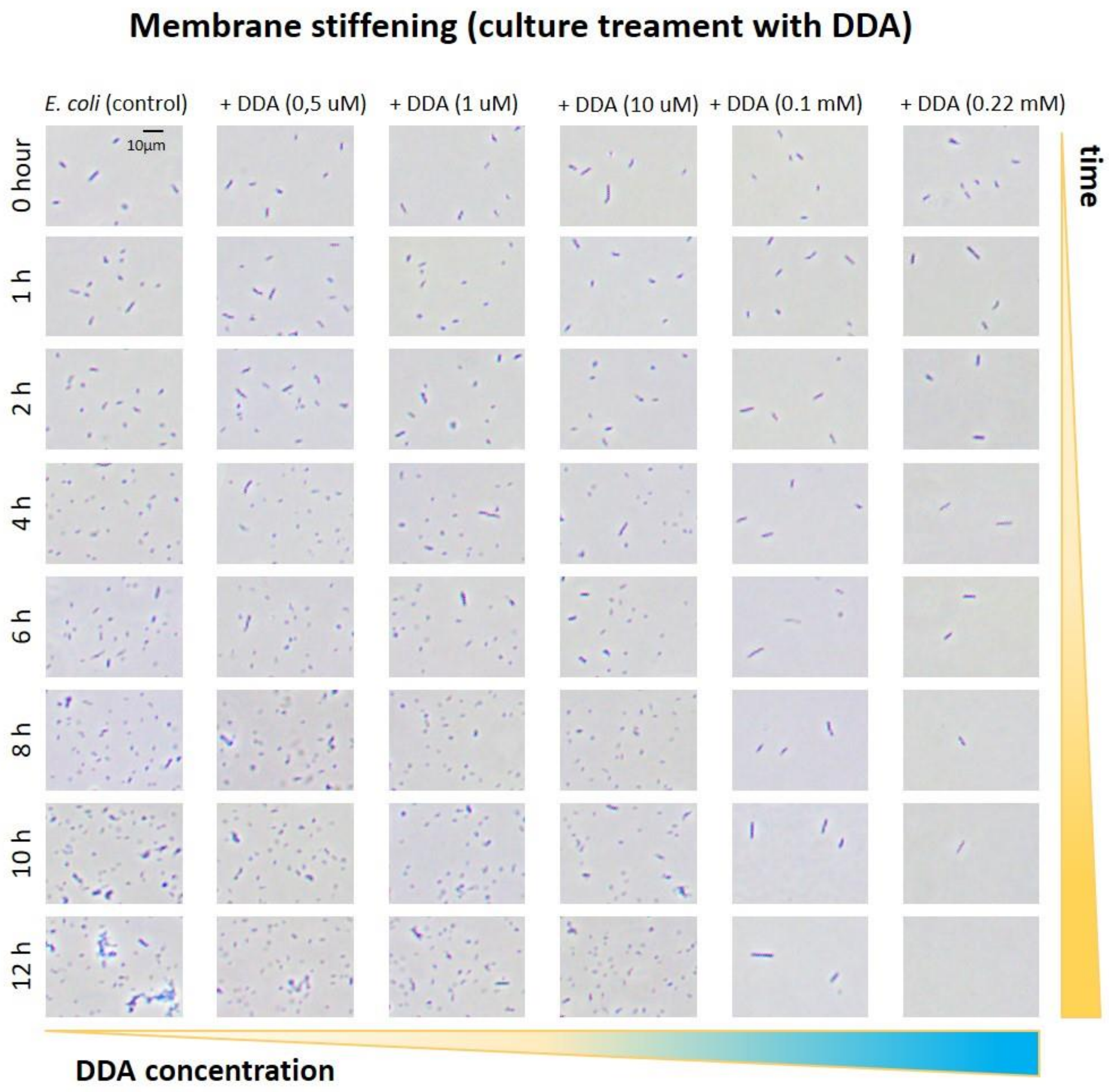

Figure S1. Photo album for E. coli cultures treated with the membrane stiffener dodecylamine (DDA). Representative photoshoot of synchronized cultures taken at advancing culture time (from top to bottom) and increasing DDA concentration (from left to right). The culture method is as described in the main paper. Micrographs taken with an inspection microscope (Nikon Eclipse Ts2, objective 20X, camera CMOS).

Supplementary Figure S2. Turbidity measurements of E. coli cultures treated with DDA

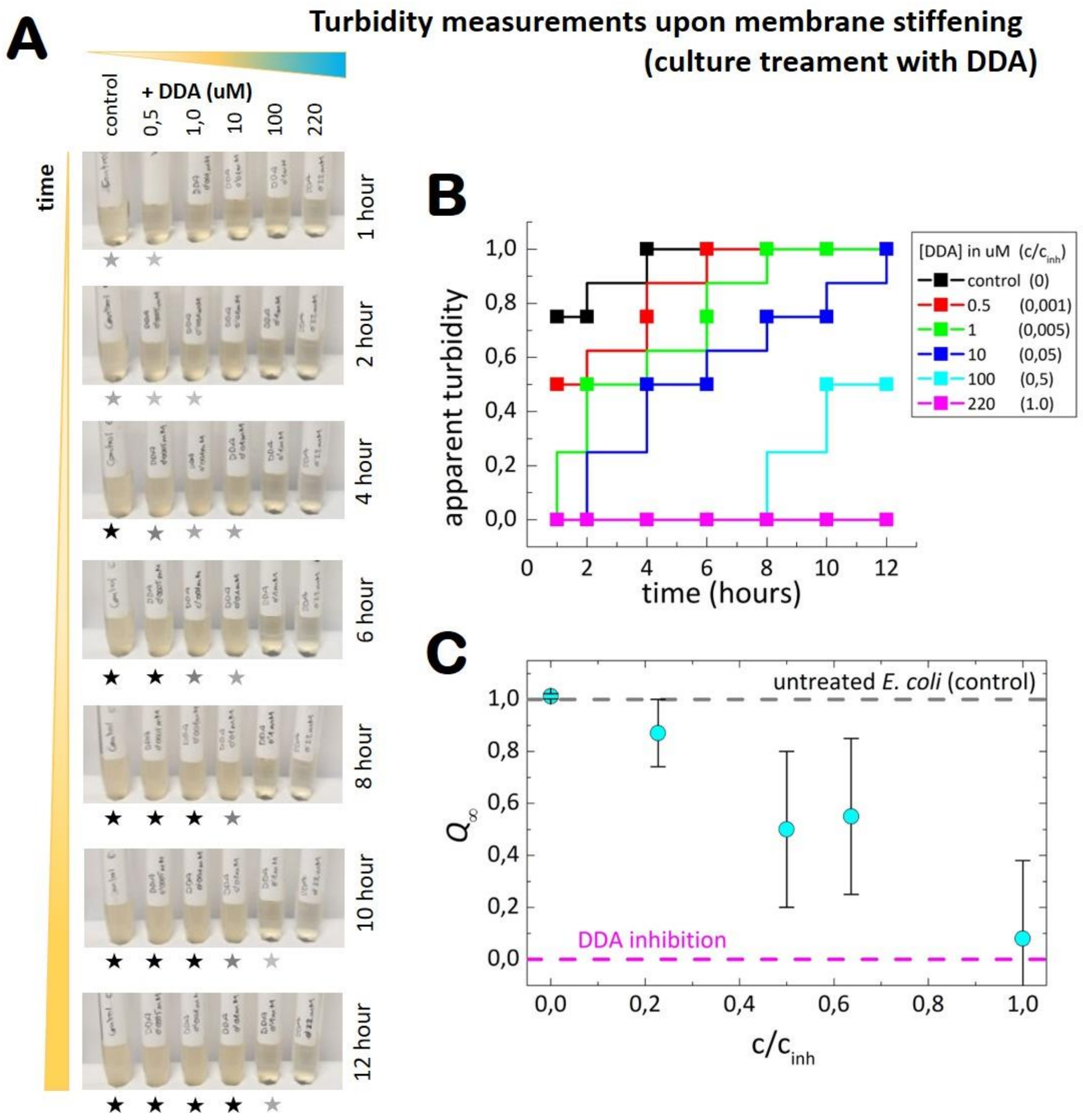

Figure S2. E. coli cultures treated with DDA in suspension. A) Sampled tubes of the synchronized cultures at advancing culture time (from top to bottom) and increasing DDA concentration (from left to right). The apparent turbidity is ranked in a qualitative scale as perceived by the naked eye (from 1 to 0: ★ 1; ★ 0.75; ★ 0.5; blank 0). **B)** Qualitative kinetic plots for different DDA concentrations (from the apparent turbidity data in panel A). An obvious decrease was observed with DDA concentration (expressed in micromolar units or referred to c_{inh} = 0.22mM). Complete inhibition was clearly detected at DDA 220 uM. **C)** Quantitative optical density (OD) at the

stationary plateau Q_∞ (as calculated from the turbidity plots in Figure 2D of the main text). The correlation with the qualitative data in B) is manifest; from the high turbidity of the control (untreated E. coli), down to the practical transparency of the culture treated with DDA at inhibitory dose.

Supplementary Figure S3. E. coli cultures treated with pentanol.

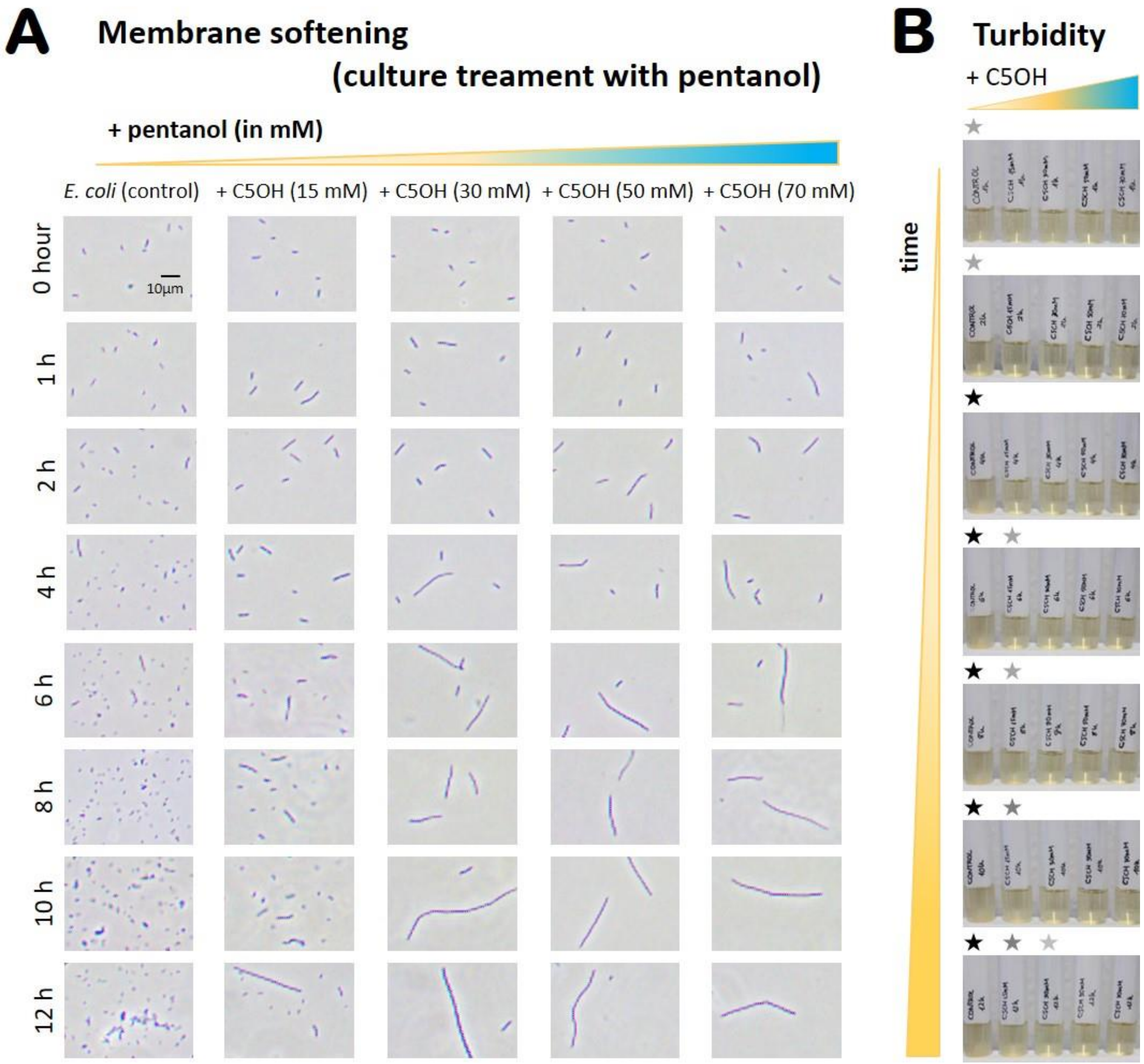

Figure S3. Photo album for E. coli cultures treated with the membrane softener pentanol (C5OH). A) Representative photoshoot of synchronized cultures taken at advancing culture time (from top to bottom) and increasing pentanol concentration (from left to right). The culture method is as described in the main paper. Micrographs taken with an inspection microscope (as in Fig. S1). For pentanol, the inhibition concentration is $c_{inh} \approx$ 90mM. **B)** Sampled tubes of the synchronized cultures at advancing culture time (from top to bottom) and increasing pentanol concentration (from

left to right). The apparent turbidity is ranked in a qualitative scale as perceived by the naked eye (as in Fig. S2).

Supplementary Figure S4. Kurtosis of the membrane fluctuation distributions in living E. coli: Softening treatment with pentanol.

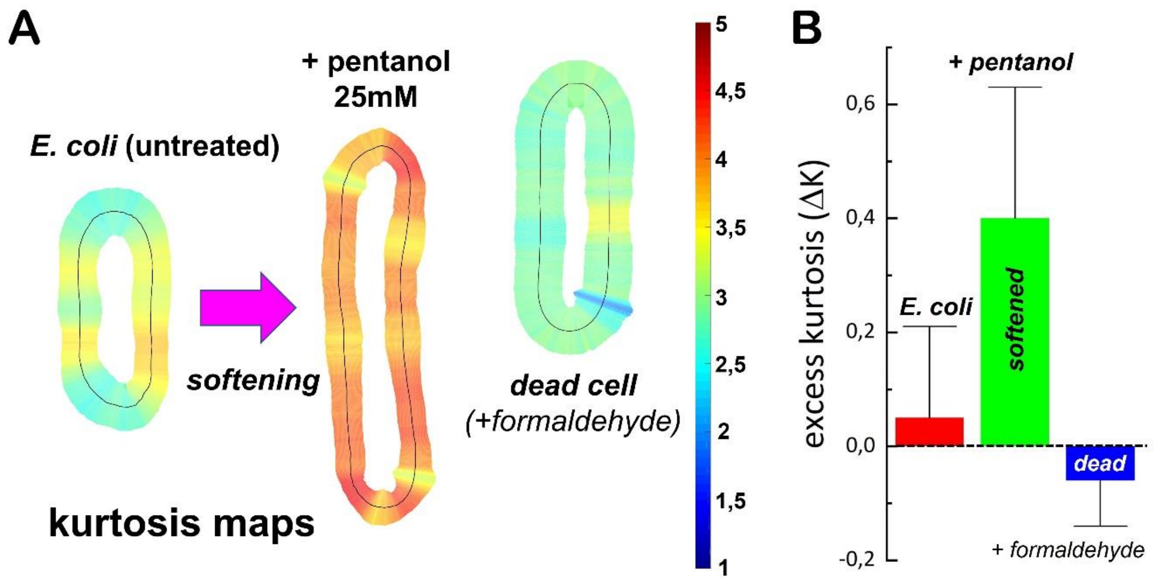

Figure S4. Membrane softening scenario at enhanced biological activity. A) Spatial maps of kurtosis (K) as calculated as the third moment of the fluctuation PDFs at each membrane emplacement. Softening treatment with pentanol caused platykurtic enhancement of the membrane fluctuations into long-tailed PDFs containing out-of-equilibrium contributions due to membrane displacements larger than expected for a pure thermal distribution (active non-Gaussianity; $K > 3$). A dead cell obtained after crosslinking treatment with formaldehyde is included for comparison (nearly Gaussian, $K \approx 3$). **B)** Statistical analysis of the excess kurtosis ($\Delta K = K - 3$), as detected in three populations of E. coli specimens ($N \geq 20$): Untreated cells (considered the control case of normal activity; in red), softened cells after treatment with pentanol (green), and dead cells after treatment with formaldehyde (considered the null hypothesis for biological activity; in blue).

Supplementary Figure S5. Kurtosis of the membrane fluctuations under cell exhaustion in E. coli.

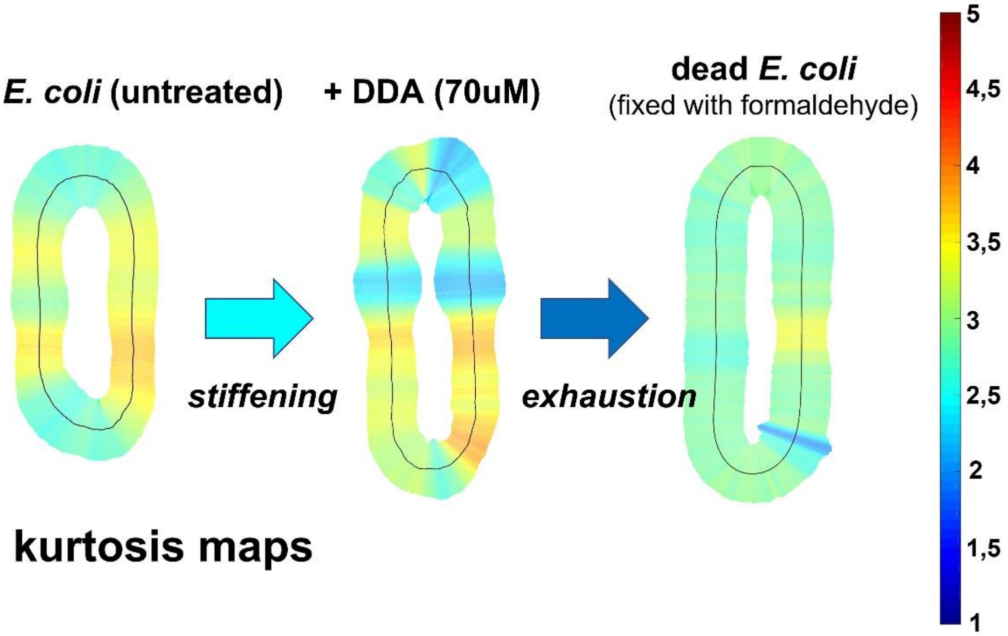

Figure S5. Membrane stiffening scenario leading biological exhaustion. Spatial maps of kurtosis (K) (see caption in Figure S4 for details). Stiffening treatment with dodecylamine (DDA) caused evident losses of the large membrane fluctuations of untreated cells ($K > 3$, characteristic of biological activity; left panel). Under DDA-stiffening, consequently, the negative excess kurtosis vanishes appearing as a progressive “cooling” at some membrane emplacements ($K \to 3$; central panel). This thermalization is evident in the dead cell as a biological exhaustion ($K \approx 3$; right panel).

Supplementary Figure S6. Complementary growth curves for E. coli cultured with propofol, nicotine and palmitic acid.

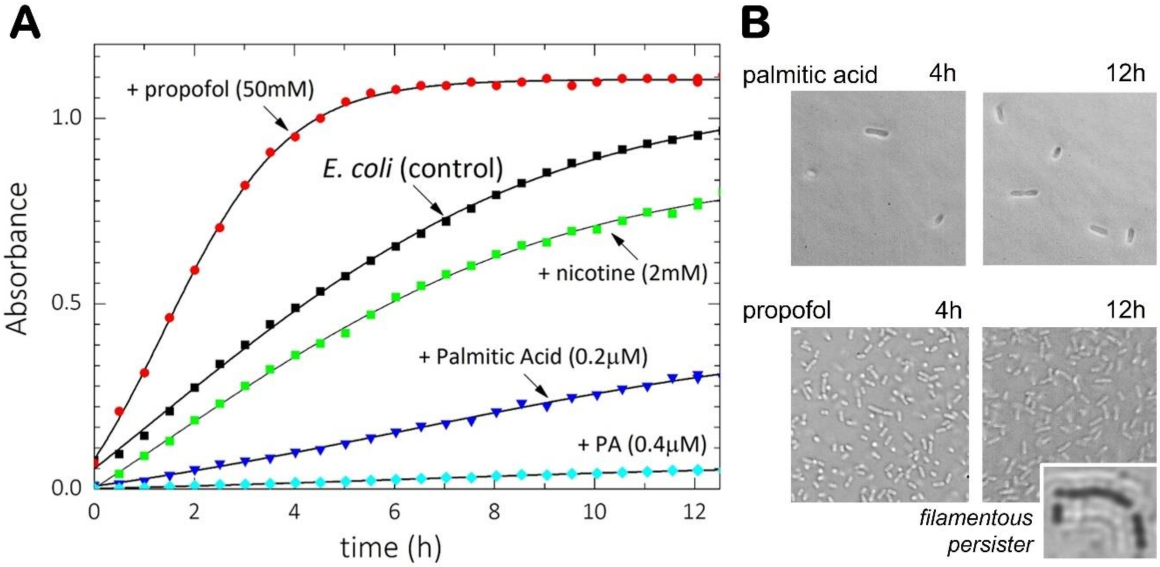

Figure S6. Complementary turbidity data. A) Kinetic growth plots of E. coli cultures in the presence of different mechano-active additives causing proliferation speeding up (propofol) and slowing down (nicotine in and palmitic acid). Propofol and nicotine are water soluble, thus they were dissolved in the same culture buffer. Palmitic acid was vehiculized in methanol. **B)** Ordinary E. coli cultures in the presence of palmitic acid as a bacteriostatic inhibitor at 0.2 μM (top panels), and propofol as a proliferation enhancer at 50 mM (bottom panels). The extraordinary appearance of short filamentous cells is indicative for adaptative propofol-dysregulation into nondividing phenotypes as rarely persistent individuals.

Supplementary Table T1.

Supplementary Table T1. Experimental values of the effective growth rates (G_{eff}) and lag times (t_0) of the bacterial cultures in Supplementary Figure S6. We also measured experimentally the compression modulus (ϵ) of the Langmuir monolayers and the bending rigidity (κ) of the giant vesicles made of E. coli PLE mixed with the corresponding additive. The compression modulus was determined at the equivalent bilayer pressure ($\pi_b = 30\, mN/m$). The bending moduli were measured by flickering spectroscopy over a population of giant vesicles ($N = 15-20$).

	**Propofol**		**Nicotine**		**Palmitic acid**	
concentration	*5 mM*	*50 mM*	*1 mM*	*2 mM*	*0.2 μM*	*0.4 μM*
$G_{eff}\ (h^{-1})$	*na*	0.85 ± 0.15	*na*	1.0 ± 0.5	0.15 ± 0.07	0.02 ± 0.01
$t_0\ (h)$	0.5 ± 0.4	0.7 ± 0.5	0 (fixed)	0 (fixed)	0 (fixed)	0 (fixed)
$\epsilon\ (mN/m)$	40 ± 10	30 ± 5	90 ± 10	100 ± 20	100 (±15%)	120 (±20%)
$\kappa\ (k_BT)$	3.0 ± 0.5	3.1 ± 0.5	5.2 ± 0.8	7.3 ± 1.0	25 (±10%)	35 (±15%)

Supplementary References

[1] R.A. Barrio, T. Alarcón and A. Hernández-Machado, The dynamics of shapes of vesicle membranes with time dependent spontaneous curvature. PLoS ONE 15: e0227562 (2020).

[2] Q. Du, C. Liu and X. Wang. A phase field approach in the numerical study of the elastic bending energy for vesicle membranes. J. Comp. Phys. 198, 450 (2004).

[3] T. Biben, K. Kassner, C. Misbah, Phase-field approach to three-dimensional vesicle dynamics. Phys. Rev. E 72, 041921 (2005).

[4] J.S. Lowengrub, A. Rätz and A. Voigt, Phase-field modeling of the dynamics of multicomponent vesicles: Spinodal decomposition, coarsening, budding and fission. Phys. Rev. E 79, 031926 (2009).

[5] C.M. Elliott, B. Stinner, Modeling and computation of two phase geometric biomembranes using surface finite elements. J. Comp. Phys. 229, 6585 (2010).

[6] F. Campelo and A. Hernández-Machado. Polymer-induced tubulation in lipid vesicles. Phys. Rev. Lett. 100, 158103 (2008).

[7] C.B. Picallo, R.A. Barrio, C. Varea, T. Alarcon and A. Hernandez-Machado. Phase-field modelling of the dynamics of Z-ring formation in liposomes: Onset of constriction and coarsening. Eur Phys J E. 38: 61–270 (2015). pmid:26105960.

[8] S. Rueda, M. Vicente and J. Mingorance. Concentration and assembly of the division ring proteins FtsZ, FtsA, and ZipA during the Escherichia coli cell cycle. J. Bacteriol. 185, 3344 (2003).

[9] H.P. Erickson, D.E. Anderson and M. Osawa. FtsZ in bacterial cytokinesis: cytoskeleton and force generator all in one. Microbiol. Mol. Biol. Rev. 74, 504 (2010).

[10] V.G. Almendro-Vedia, P. Natale, M. Mell, S. Bonneau, F. Monroy, F. Joubert and I. López-Montero. Nonequilibrium fluctuations of lipid membranes by the rotating motor protein F1F0-ATP synthase. Proc. Nat. Acad. Sci. USA 114, 11291 (2017).

[11] M. Mell and F. Monroy. A gradient-based, GPU-accelerated, high-precision contour segmentation algorithm with application to cell membrane fluctuation spectroscopy. PloS One 13, e0207376 (2018).

[12] J. Pécreaux, H.G. Döbereiner, J. Prost, J.F. Joanny and P. Bassereau. Refined contour analysis of giant unilamellar vesicles. Eur. Phys. J. E 13, 277 (2004).

[13] L.R. Arriaga, I. López-Montero, G. Orts-Gil, B. Farago, T. Hellweg, F. Monroy, Fluctuation dynamics of spherical vesicles: Frustration of regular bulk dissipation into subdiffusive relaxation. Phys. Rev. E 80, 031908 (2009).

[14] L.R. Arriaga, R. Rodríguez-García, I. López-Montero, B. Farago, T. Hellweg and F. Monroy. Dissipative curvature fluctuations in bilayer vesicles: Coexistence of pure-bending and hybrid curvature-compression modes. Eur. Phys J. E 31, 105 (2010).

[15] R. Rodríguez-García, L.R. Arriaga, M. Mell, L.H. Moleiro, I. López-Montero and F. Monroy. Bimodal spectrum for the curvature fluctuations of bilayer vesicles: Pure bending plus hybrid curvature-dilation modes. Phys. Rev. Lett. 102, 128101 (2009).

[16] H.A. Faizi, S.L. Frey, J. Steinkühler, R. Dimova and P.M. Vlahovska. Bending rigidity of charged lipid bilayer membranes. Soft Matter 15, 6006, 128101 (2019).

[17] E. Evans and D. Needham. Physical properties of surfactant bilayer membranes: thermal transitions, elasticity, rigidity, cohesion and colloidal interactions. J. Phys. Chem. 91, 4219 (1986).

[18] R. Dimova. Recent developments in the field of bending rigidity measurements on membranes. Adv. Colloid Interf. Sci. 208, 225 (2014).

[19] M. Mell, L.H. Moleiro, Y. Hertle, I. López-Montero, F.J. Cao, P. Fouquet, T. Hellweg and F. Monroy. Fluctuation dynamics of bilayer vesicles with intermonolayer sliding: Experiment and theory. Chem. Phys. Lipids 185, 61 (2015).

[20] J.F. Nagle, M.S. Jablin, S. Tristram-Nagle and K. Akabori. What are the true values of the bending modulus of simple lipid bilayers? Chem. Phys. Lipids 185, 3 (2015).

[21] M. Mell, L.H. Moleiro, Y. Hertle, P. Fouquet, R. Schweins, I. López-Montero and F. Monroy. Bending stiffness of biological membranes: What can be measured by neutron spin echo? Eur. Phys. J. E 36, 75 (2013).

[22] D. Marsh. Elastic curvature constants of lipid monolayers and bilayers. Chem. Phys. Lipids 144, 146 (2006).

[23] D.H. Boal, Mechanics of the cell, 2nd Ed. (Cambridge University Press, Cambridge, 2012).

[24] J. M. Seddon, R. H. Templer, N. A. Warrender, Z. Huang, G. Cevc and D. Marsh. Phosphatidylcholine–fatty acid membranes: effects of headgroup hydration on the phase behaviour and structural parameters of the gel and inverse hexagonal (HII) phases. Biochim. Biophys. Acta - Biomembranes 1327, 131 (1997).

[25] A.H. Hansen et al. Propofol modulates the lipid phase transition and localizes near the headgroup of membranes. Chem. Phys. Lipids 175-176, 84 (2013).

[26] H. Bouvrais, P. Méléard, T. Pott, K.J. Jensen, J. Brask and J. H. Ipsen. Softening of POPC membranes by magainin. Biophys. Chem. 137, 7 (2008).

[27] H. I. Ingolfsson and O.S. Andersen. Alcohol's effects on lipid bilayer properties. Biophys. J. 101, 847 (2011).

[28] W. Helfrich, Blocked lipid exchange in bilayers and its possible influence on the shape of vesicles. Z. Naturfosch 29c, 510 (1974).

[29] A. Yeung and E. Evans. Unexpected dynamics in shape fluctuations of bilayer vesicles. J. Phys. II 5, 1501 (1995).

[30] P.L. Hansen, L. Miao and J.H. Ipsen. Fluid lipid bilayers: Intermonolayer coupling and its thermodynamic manifestations. Phys. Rev. E 58, 2311 (1998).

[31] W. Rawicz, K.C. Olbrich, T. McIntosh, D. Needham and E. Evans. Effect of chain length and unsaturation on elasticity of lipid bilayers. Biophys. J. 79, 328 (2000).

[32] P. Shchelokovskyy, S. Tristram-Nagle and R. Dimova. Effect of Effect of the HIV-1 fusion peptide on the mechanical properties and leaflet coupling of lipid bilayers. New J. Phys. 13, 025004 (2011).

[33] M.A. Bahri, B.J. Heyne, P. Hans, A.E. Seret, A.A. Mouithys-Mickalad and M.D. Hoebeke. Quantification of lipid bilayer effective microviscosity and fluidity effect induced by propofol. Biophys. Chem. 114, 53 (2005).

[34] W. Shinoda. Permeability across lipid membranes. Biochim. Biophys. Acta – Biomembranes 1858, 2254 (2016).

[35] A. Khondker, A. Dhaliwal, R.J. Alsop and J. Tang. Partitioning of caffeine in lipid bilayers reduces membrane fluidity and increases membrane thickness. Phys. Chem. Chem. Phys. 19, 7101 (2017).

[36] Cholesterol in Membrane Models by L. X. Finegold (1992, CRC Press)

[37] T. Róg, M. Pasenkiewicz-Gierula, I. Vattulainen and M. Karttunen. Ordering effects of cholesterol and its analogues. Biochim. Biophys. Acta - Biomembranes. 1788, 97-121 (2009).

[38] S. Cooper and C.E. Helmstetter, Chromosome replication and the division cycle of Escherichia coli B/r. J. Mol. Biol. 31, 619 (1968).

[39] A. Zaritsky and R. H. Pritchard, Changes in cell size and shape associated with changes in the replication time of the chromosome of Escherichia coli, J. Bacteriol. 114, 824 (1973).

[40] H. Bremer and P.P. Dennis, Modulation of chemical composition and other parameters of the cell at different exponential growth rates, Ecosal Plus 3 (2008).

[41] M. Scott, C.W. Gunderson, E.M. Mateescu, Z. Zhang and T. Hwa, Interdependence of cell growth and gene expression: origins and consequences, Science 330 1099 (2010).

[42] F.C. Neidhardt, Bacterial growth: constant obsession with dN/dt. J. Bacteriol. 181, 7405 (1999).

[43] M. Schaechter, O. Maaløe and N.O. Kjeldgaard, Dependency on medium and temperature of cell size and chemical composition during balanced grown of Salmonella typhimurium, J. Gen. Microbiol. 19, 592 (1958).

[44] F. Si et al., Invariance of initiation mass and predictability of cell size in Escherichia coli, Curr. Biol. 27, 1278 (2017).

[45] B.P. Hills and K.M. Wright. A new model for bacterial growth in heterogeneous systems. J. Theor. Biol. 168(1), 31 (1994).

[46] P. Usenik, T. Vrtovec, F. Pernuš and B. Likar. Automated tracking and analysis of phospholipid vesicle contours in phase contrast microscopy images. Med. Biol. Eng. Comput. 49:957 (2011).

[47] J. Pécréaux, H.G. Döbereiner, J. Prost, J.F. Joanny and P. Bassereau. Refined contour analysis of giant unilamellar vesicles. Eur. Phys. J. E 13:277 (2004).

[48] X. Ma, D.W. Ehrhardt and W. Margolin. Colocalization of cell division proteins FtsZ and FtsA to cytoskeletal structures in living Escherichia coli cells by using green fluorescent protein. Proc Natl Acad Sci USA. 93:12998 (1996).

[49] B.P. Hills and K.M. Wright. A new model for bacterial growth in heterogeneous systems. J. Theor. Biol. 168, 31 (1994).
